# Supplementary figures and images for: Provision of preferred nutrients to macrophages promotes Salmonella intracellular replication without relying on Type III secretion systems
Source: PLoS Pathog. 2026 Jul 9;22(7):e1014348. doi: 10.1371/journal.ppat.1014348 (PMC13349091; doi:10.1371/journal.ppat.1014348)

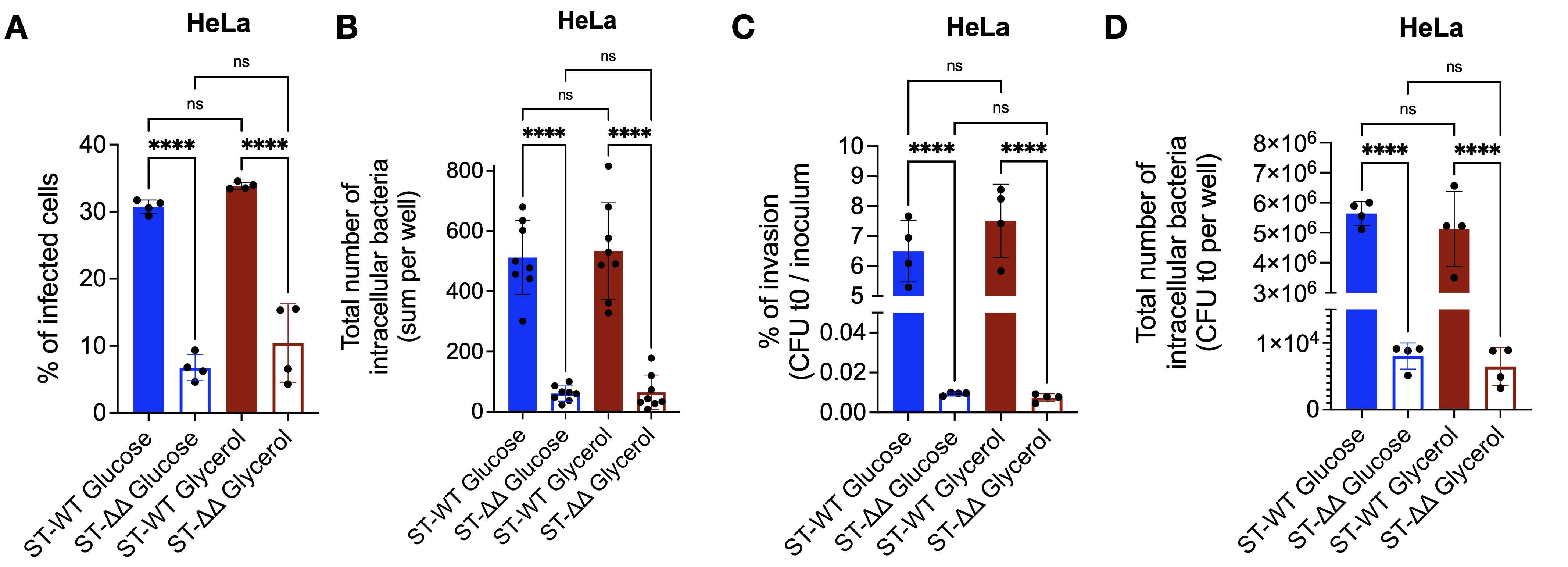

Supplement: S2 Fig — Related to S1G Fig. HeLa cells were cultured in nutrient-limited medium supplemented with 10 mM glucose or 10 mM glycerol and infected with ST-WT or the T3SS-deficient ST-ΔprgH/ΔssaV strain (ST-ΔΔ) at an MOI of 50. (A) Percentage of infected HeLa cells determined by automated confocal microscopy. (B) Total number of intracellular bacteria per well determined by automated confocal microscopy. (C) Percentage of bacterial invasion measured by gentamicin protection assay, calculated as the number of intracellular colony-forming units (CFU) recovered at t0 per well divided by the inoculum per well (CFUt0/inoculum). (D) Total number of intracellular bacteria per well measured by gentamicin protection assay (CFUt0 per well). Bars represent the mean ± SD and each dot represents an independent replicate. Statistical comparisons were performed by ordinary one-way ANOVA (**** = p < 0.0001, ns = not significant). (TIFF) [file ppat.1014348.s002.tiff]

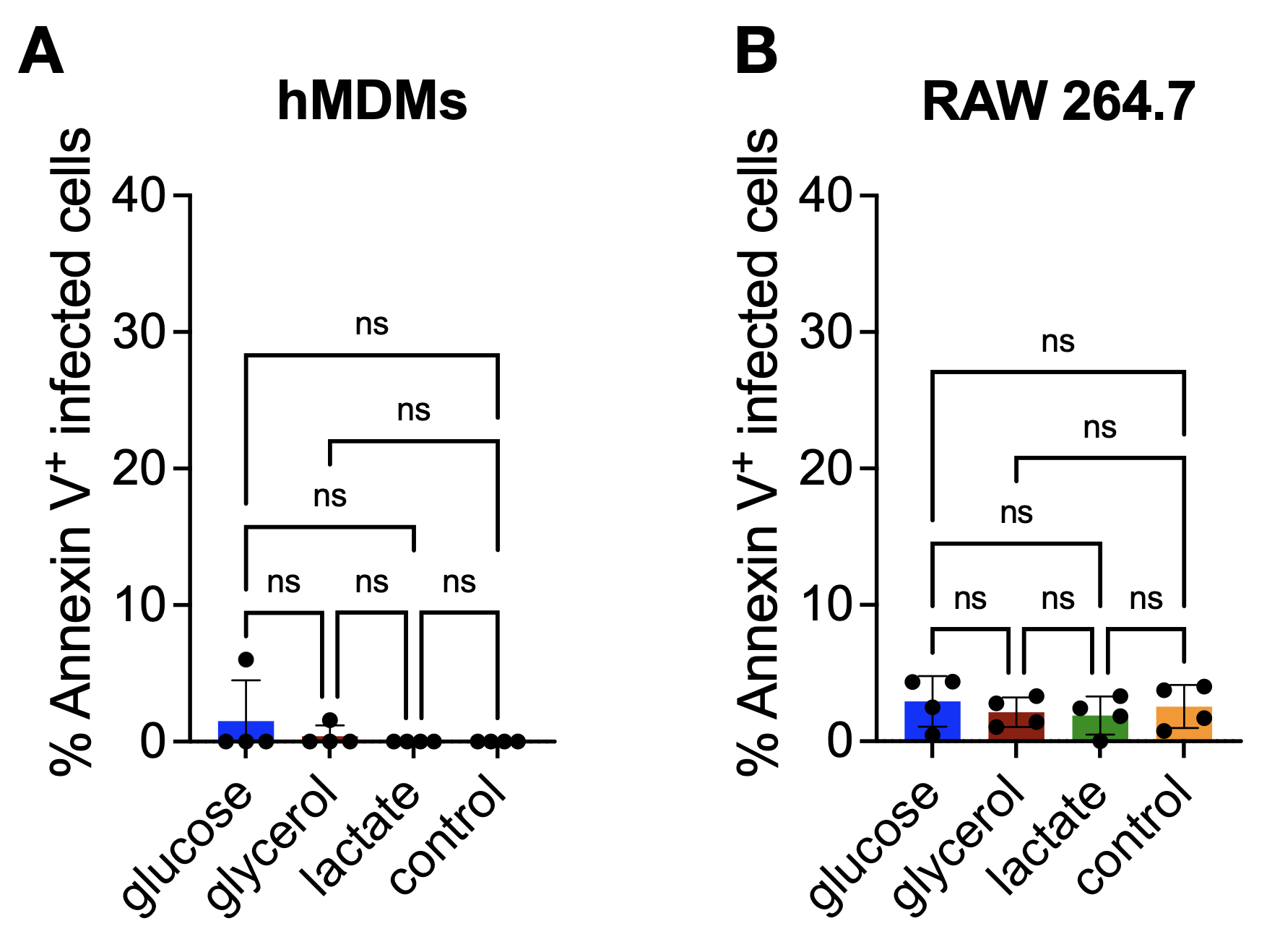

Supplement: S3 Fig — (A) hMDMs cultured in nutrient-limited media supplemented with different carbon sources (10 mM) were infected with ST-WT. AnnexinV Alexa Fluor 647 was added to the cell culture to monitor early cell death during infection. Bars show the percentage of infected cells that were AnnexinV+ cells at 8 hours post-infection. (B) Same as (A) in RAW 264.7 macrophages infected with ST-WT. Bars represent the mean ± SD of four replicates from one experiment, representative of three independent experiments. (ns = not significant, ordinary one-way ANOVA). (TIFF) [file ppat.1014348.s003.tiff]
