# Supplementary material for: Provision of preferred nutrients to macrophages promotes Salmonella intracellular replication without relying on Type III secretion systems
Source: PLoS Pathog. 2026 Jul 9;22(7):e1014348. doi: 10.1371/journal.ppat.1014348 (PMC13349091; doi:10.1371/journal.ppat.1014348)
Supplement: Attachment — Submitted filename: Revision-plan-RC-2025-03098.pdf [file ppat.1014348.s006.pdf]

# Revision Plan

Review COMMONS

**Manuscript number:** RC-2025-03098
**Corresponding author:** Pedro Escoll

## 1. General Statements

Our study investigates the interplay between the metabolism of host cells and the intracellular replication of *Salmonella enterica* serovar Typhimurium (ST). Type III Secretion Systems (T3SSs) are traditionally considered essential for ST to survive and replicate within macrophages. However, we found that restricting macrophages to different bioenergetic contexts, such as supplementing them with glycerol, modulates bacterial replication and remarkably, enables a T3SS-deficient ST mutant (Δ*prgH*/Δ*ssaV*) to replicate intracellularly. This T3SS-independent replication occurs within the *Salmonella*-containing vacuole (SCV) and is driven by the capacity of the host cell to provide these preferred nutrients, rather than by the host glycolytic activity itself.

## 2. Description of the planned revisions

**Reviewer #1 (Evidence, reproducibility and clarity):**

Summary:

In this manuscript, the authors investigate how host cell metabolic heterogeneity influences the intracellular replication of *Salmonella enterica* serovar Typhimurium. They use live-cell imaging of infected human primary macrophages to reveal that bacterial replication does not occur uniformly across infected cells. They demonstrate that supplementation with specific carbon sources-used by *Salmonella* during infection-promotes bacterial replication and increases the proportion of macrophages supporting intracellular growth. These effects are seen even in the absence of functional Type III Secretion Systems (T3SS), using a Δ*prgH*/Δ*ssaV* double mutant. The authors further suggest that this replication enhancement is not strictly dependent on host glycolytic activity but rather on the host cell's ability to import nutrients. Their findings imply that intracellular *Salmonella* can exploit host cell metabolism to grow, even without its canonical virulence secretion systems, under nutrient-favorable conditions.

Major Concern:

While the topic is potentially interesting, the novelty is not fully clear. The concept that nutrient availability impacts intracellular *Salmonella* replication, largely via T3SS2 function, has been addressed previously (e.g., Liss et al., 2017). The finding that added exogenous carbon sources can enhance bacterial growth is thus not unexpected. The key claim-that *Salmonella* can replicate intracellularly even in the absence of T3SS function-would be significantly

strengthened by demonstrating whether this is specific to *Salmonella*, or whether similar effects are seen with non-intracellular organisms such as *E. coli* K-12. If the phenomenon is unique to *Salmonella*, this would suggest a pathogen-specific mechanism beyond general metabolic support.

As acknowledged by the Reviewer, the novelty and key claim of our work is that *Salmonella* can replicate intracellularly even in the absence of T3SS. This is clearly stated in the title of the manuscript. To experimentally sustain that claim, we showed evidence that providing macrophages with the preferred carbon sources used by *Salmonella* during infection, such as glycerol, bypass the requirement of both T3SS by *Salmonella* to grow, intravacuolarly, inside macrophages.

Regarding the key article mentioned by the Reviewer (Liss *et al.* 2017, ref 36 in the manuscript), there are three important novel insights: *i)* we show that *Salmonella* can replicate intracellularly in the SCV even in the absence of T3SS if certain carbon sources are provided; *ii)* we show the preference of *Salmonella* for certain carbon sources intracellularly such as glycerol and galactose (but not preferentially glucose); and *iii)* we have extended our observations to primary human macrophages too in addition to RAW cells.

The experiment suggested by the Reviewer of using E. coli K12 (ECK12) is very interesting and we will do it. Briefly, we will infect hMDMs and RAW macrophages with ST-WT-GFP, ST-Δ*prgH*Δ*ssaV* or ECK12-WT-GFP, while culturing macrophages on different carbon sources (glucose, glycerol, galactose, fructose). Then we will monitor intracellular bacterial growth. By comparing bacterial growth of ST double mutant with ECK12-WT-GFP under farovable carbon sources such as glycerol, the results will be definitive to answer whether this phenomeon is unique to *Salmonella* or not.

Specific Comments:

1. Figure 1H: The effect shown here is not compelling due to inconsistent y-axis scaling. Panels 1B, 1C, and 1D should use a unified axis range with 1H to allow direct visual comparison of growth dynamics.

Thank you, we will change it as suggested.

2. Figures 1B, 1C, 1G, 1H: The current presentation of individual growth traces makes it difficult to appreciate the population-level trend. A smoothed average line overlaid on these plots could better represent the average dynamics of replicative vs. non-replicative infections. Or alternatively the total fraction of cells that proliferate summarized as a segmented bar plot (possibly binned per time point).

Yes, we are going to plot the results as suggested, the total fraction of infected cells harboring bacteria that proliferate as a segmented bar plot, binned per time point.

3. Figure 2G: This panel would benefit from including a comparable condition with the SPI-1/SPI-2 double mutant to aid interpretation. Additionally, the authors should explore whether this nutrient-supported replication is seen in non-phagocytic cells such as HeLa or Caco-2, which would help delineate whether the observed phenomenon is macrophage-specific.

The graph asked by Reviewer is Figure S1D. As we are representing ST growth in macrophages supporting *Salmonella* replication, some of the conditions, such as lactate, cannot be shown in the infection conditions using the double mutant because there are no cells supporting the replication of the double mutant, so there are no cells to plot.

As suggested, we are also going to perform the same experiments in HeLa cells to know whether the observed phenomenon is macrophage-specific.

4. Line 117: The sentence stating that the double mutant can undergo “exponential intracellular growth even in the absence of T3SS-dependent secretion” is an overstatement. The data suggest only a modest improvement in growth, restricted to a minority of infected cells. This claim should be revised accordingly, as should similar overstatements in the discussion (e.g., lines 203-204).

Thank you, we agree. We will remove the term ‘exponential’ and revise the sentence at line 117 and those in the discussion. Line 203-204 will be: ‘we demonstrated that providing macrophages with preferred nutrients allows a subpopulation of ST to replicate intracellularly without the need for functional T3SS’.

5. Line 162: The authors should clarify that glycerol had the strongest effect in primary macrophages, while multiple alternative carbon sources had notable effects primarily in RAW cells.

Thank you very much for this suggestion, we will add this clarification in the text.

6. Lines 198-201: This relates to the major concern. The authors should assess whether the observed growth enhancement is unique to *Salmonella* by testing other bacteria not known for intracellular replication. This would clarify whether the effect is due to general nutrient-driven host cell permissivity or a pathogen-specific adaptation.

As said before, we will perform the suggested experiment with E. coli K12 to answer whether this phenomenon is unique to *Salmonella* or not.

7. RAW 264.7 Observations: The modest intracellular growth of SPI-1/SPI-2 double mutants in RAW cells is consistent with prior observations in the field. The idea that nutrient availability explains this is noteworthy. The authors might consider whether differences in standard culture media (e.g., glucose concentration) influence these outcomes. This could have broader implications for reproducibility in infection models.

Thank you for the suggestion, we will include a paragraph discussing whether differences in standard culture media might influence bacterial replication. Indeed, to answer also a question from Reviewer #2, we are going to include a new supplementary Figure comparing no Glucose (0 mM), low Glucose (2 mM) and standard culture media Glucose levels (10 mM), showing that differences in Glucose levels in the culture media used

influence *Salmonella* intracellular growth in hMDMs and RAW macrophages (see Figure below).

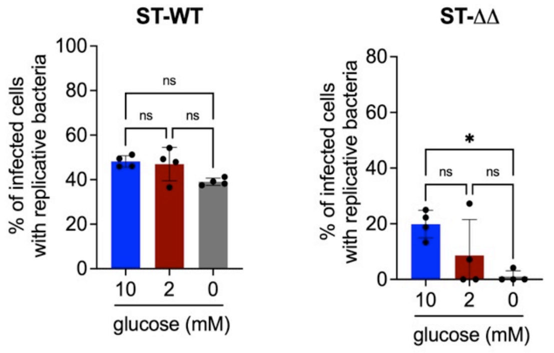


**Future Figure S2E-F. Gradual effect of glucose supplementation on T3SS-independent intracellular replication of ST in RAW 264.7 cells.** (E) Percentage of ST-WT-infected RAW 264.7 cells containing replicative bacteria when cultured in nutrient-limited medium supplemented with 10 mM, 2 mM or 0 mM of glucose. (F) Same as (A) but in RAW 264.7 cells infected with ST-Δ*prgH*/Δ*ssaV* strain (ST-ΔΔ). Bars represent the mean ± SD of four replicates from one experiment, representative of three independent experiments. (*=p<0.05, ns=non-significative, ordinary one-way ANOVA).

Reviewer #1 (Significance):

This manuscript highlights how host cell metabolism and nutrient availability can influence intracellular *Salmonella* replication. While the findings are intriguing, the current framing overstates their novelty and impact. Key revisions-such as comparative experiments with non-pathogenic bacteria and non-phagocytic cells, consistent figure scaling, and more measured language-would improve the clarity and significance of the work. If the authors can show *Salmonella*-specific mechanisms at play, the study could offer important insights into host-pathogen metabolic interactions.

We believe that performing all experiments suggested by the Reviewers, as well as the requested changes in the text to avoid the pointed overstatements, will improve the manuscript and will offer readers new insights and details to better understand the metabolic interactions happening between host and pathogens and how they can shape bacterial virulence.

**Reviewer #2 (Evidence, reproducibility and clarity):**

Summary:
In their study titled "Provision of Preferred Nutrients to Macrophages Enables *Salmonella* to Replicate Intracellularly Without Relying on Type III Secretion Systems", Dr. Garcia-Rodriguez *et al*. describe the influence of the host cell metabolism on the intracellular proliferation potential of *Salmonella* during infection. The authors investigate whether the supplementation of the media with different carbon sources has an impact on the intracellular lifestyle of *Salmonella*. By using single cell tracking in live-cell microscopy, including the use of different reporter strains, they describe that glycerol benefits *Salmonella*'s ability to grow within its vacuolar niche, in part, interestingly, in a Type-3-Secretion System independent manner.

They furthermore highlight the dependence on host background for this observation by showing that effects differ between cells of varying metabolic activity. Throughout their study, they use cutting-edge methodologies, as well as *Salmonella* strains that could be of versatile use in other investigations. This work, while limited to *in vitro* models for now, has implications for the better understanding of how pathogens and their host are intertwined. This, in turn, has significance for the development of new anti-infective strategies further down the line. I therefore believe that it should be disseminated to the research community. The following comments summarize ideas how the quality of the study could be improved:

Major comments:

1. *Salmonella*, especially when cultured to activate the SPI-1 T3SS, introduce rapid cell death in their host - most commonly through activation of the NLRC4 inflammasome and downstream pyroptotic signaling. The authors don't describe the effect of the infection in differently supplemented media on host cell death, yet it would be important to elucidate whether this cellular response is also altered.

> We have performed these experiments and tracked host cell death by measuring Annexin-V levels in single cells, during infection in the conditions using the different supplements. We will include these results in the revised version of the manuscript and main text. Please see the Figure below showing that supplementations did not affect significantly macrophages cell death (future Figure S1E and S1F)

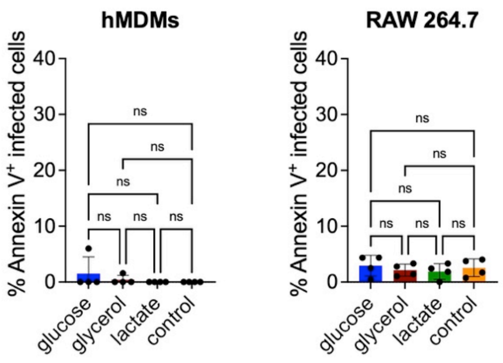


**Future Figure S1E-F.**
**Carbon source supplementation does not affect host cell death. (E)** hMDMs cultured in nutrient-limited media supplemented with different carbon sources (10 mM) were infected with ST-WT. AnnexinV Alexa Fluor 647 was added to the cell culture to monitor early cell death during infection. Bars show the percentage of infected cells that were AnnexinV+ cells at 8 hours post-infection. **(F)** Same as **(E)** in RAW 264.7 macrophages infected with ST-WT. Bars represent the mean ± SD of four replicates from one experiment, representative of three independent experiments. (ns=non-significative, ordinary one-way ANOVA).

2. The aspect of partially T3SS-independent growth enhancement by glycerol (and depending on the host background glucose) is most curious. The authors quantify this by determining the percentage of cells containing proliferating *Salmonella* and by tracking individual cells over the time course of the infection. I am missing a general statement on whether the initial infection rate (*i.e.* timepoint 0) is comparable across conditions and mutants, and whether possible discrepancies in the infection rate could have downstream effects on the statements and claims made in the manuscript. This is, to my mind, also important for the quantification of cytosolic and vacuolar bacteria. There, the authors always speak in "percent of infected cells", so it is relevant whether the number of infected cells varies among conditions (see *e.g*. Figure 3).

> We thank the reviewer for this comment. The initial infection rate at t=0 significantly differs between WT and mutants in RAW 264.7 macrophages, and carbon source supplementation has no effect. However, as we only analyze infected cells, this does not affect the final results. In any case, we are going to add the graphs of % of infected cells at t=0 as supplementary Figures S1G-K.

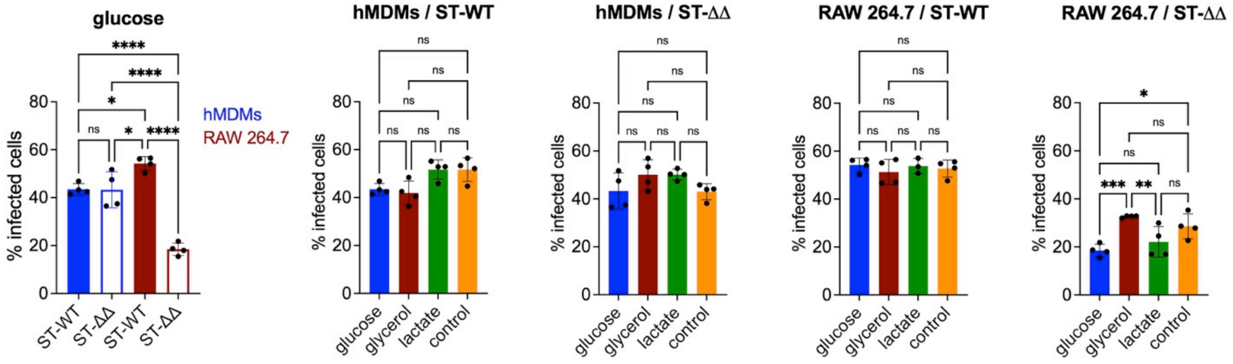


**Future Figure S1G-K. Impact of ST strain and carbon source supplementation on infection efficiency in macrophages. (G)** Percentage of infected cells at T=0 of hMDMs and RAW 264.7 cells, cultured in nutrient limited medium supplemented with 10 mM glucose and infected with ST-WT or ST-ΔprgH/ΔssaV strain (ST-ΔΔ) at 20 hpi. **(H)** Percentage of ST-WT infected hMDMs at T=0 under different carbon source supplementation conditions (10 mM). **(I)** Same as (H) in hMDMs infected with ST-ΔΔ strain. **(J)** Same as (H) in RAW 264.7 macrophages infected with ST-WT. **(K)** Same as (H) in RAW 264.7 cells infected with ST-ΔΔ strain. Bars represent the mean ± SD of four replicates from one experiment, representative of three independent experiments. (*=p<0.05 **p < 0.01, ***p < 0.001, ns=non-significative, ordinary one-way ANOVA).

3. The authors use a concentration of 10mM for all supplemented alternative carbon sources. It would be useful to discuss the rationale behind this approach, including whether all chemicals have the same ability to be taken up by the cell. A concentration series (at least for some of the tested compounds) may be beneficial to bolster the conclusions that the authors make.

> We use 10 mM as this is the concentration of Glucose in standard culture media. By using 10 mM for all the different carbon sources, we can thus compare them keeping concentration constant (10 mM). Indeed, to answer also Reviewer #1, we will include in the manuscript a paragraph discussing whether differences in standard culture media might influence bacterial replication. As this Reviewer suggested, we will include a new supplementary Figure comparing no Glucose (0 mM), low Glucose (2 mM) and standard

culture media Glucose levels (10 mM), showing that the concentration of glucose has a gradual effect in supporting the replication of the T3SS-deficient strain in RAW macrophages (see Figure below).

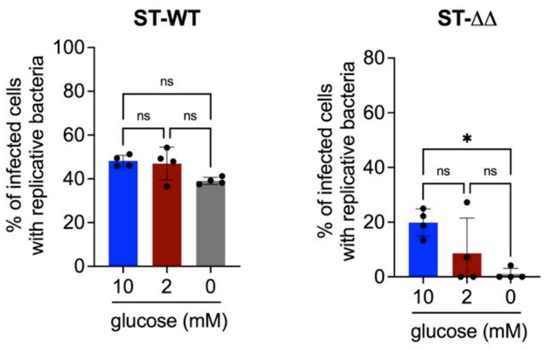


**Future Figure S2E-F. Gradual effect of glucose supplementation on T3SS-independent intracellular replication of ST in RAW 264.7 cells. (E)** Percentage of ST-WT-infected RAW 264.7 cells containing replicative bacteria when cultured in nutrient-limited medium supplemented with 10 mM, 2 mM or 0 mM of glucose. **(F)** Same as (A) but in RAW 264.7 cells infected with ST-Δ*prgH*/Δ*ssaV* strain (ST-ΔΔ). Bars represent the mean ± SD of four replicates from one experiment, representative of three independent experiments. (*=p<0.05, ns=non-significative, ordinary one-way ANOVA).

4. I think it would strengthen the study, if th metabolite transporters, or alternatively *Salmonella* mutants that are deficient in uptake or metabolism of some of the compounds used in this study. This point is alluded to in the discussion, and I believe if the authors could show that in certain host mutant backgrounds the impact of supplementation with alternative carbon sources can be reversed, it would immensely bolster the strength of the claims.

Following Reviewer's suggestion, we generated ST metabolic mutants unable to metabolize glycerol, galactose or fructose. As can be seen in the Figures below, during infection, the supplementations with glycerol/galactose does not boost *Salmonella* replication in metabolic mutants as in WT conditions, demonstrating that supplemented carbon sources indeed arrive to bacteria within the SCV and are used by intracellular *Salmonella* to grow. This Figures will be now Future Figure 4J-N.

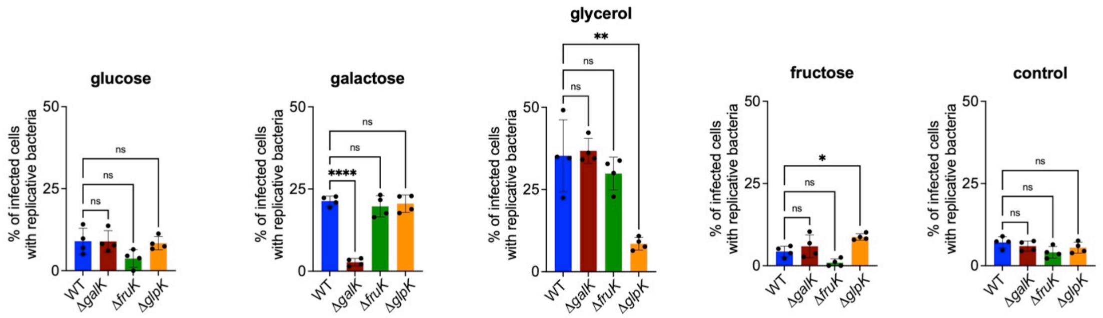


**Future Figures 4J-N. Intracellular replication promoted by glycerol or galactose supplementation is impaired in ST mutants unable to metabolize these carbon sources. (J)** hMDMs were infected with ST-WT or ST metabolic mutants unable to metabolize galactose (Δ*galK*), fructose (Δ*fruK*), or glycerol (Δ*glpK*). The percentage of infected hMDMs containing replicative bacteria was measured in nutrient-limited medium supplemented with 10 mM glucose. **(K)** Same as (J) but supplemented with 10 mM galactose. **(L)** Same as (J) but supplemented with 10 mM glycerol. **(M)** Same as (J) but supplemented with 10 mM glycerol. **(N)** same as (J) but in non-supplemented media served as a control. Bars represent the mean ± SD of four replicates from one experiment, representative of two independent experiments with two different donors. (*=p<0.05 **p < 0.01, ****p < 0.0001, ns=non-significative, ordinary one-way ANOVA).

5.
in
the pathogens are at hand, it might be interesting to infect with other intracellular bacteria, such

as *Shigella* or *Francisella* to investigate if the boosting of growth by glycerol also holds true for these.

We have performed experiments with *Legionella pneumophila* and galactose (see figure below), showing that this carbon source is in this case specific of *Salmonella* (as shown in Figure 4F in the manuscript). We are going to perform experiments also with *L. pneumophila* and glycerol to satisfy the interest of the Reviewer. However, we think that the results with *Legionella* might be out of the focus of this article and would constitute themselves a new article, as both pathogens have a very different, non-comparable intracellular metabolism. For the revision, we are going to perform the experiment suggested by Reviewer #1 of using E. coli K12 (ECK12) while culturing macrophages on different carbon sources (glucose, glycerol, galactose, fructose). We will monitor intracellular bacterial growth and, by comparing bacterial growth of the ST-Δ*prgH*/Δ*ssaV* double mutant with ECK12-WT-GFP under favorable carbon sources such as glycerol, the results will be definitive to answer whether this phenomenon is unique to *Salmonella* or not.

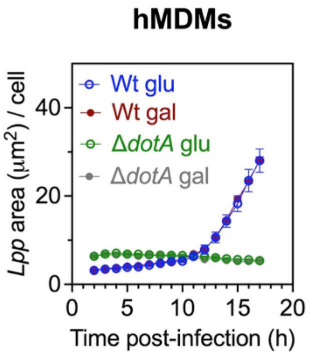


**Figure R1. Only for the Reviewer. Galactose supplementation does not enhance intracellular growth of *Legionella pneumophila* in hMDMs.** Intracellular growth dynamics of *Legionella pneumophila* strain Paris wild-type strain (*Lpp*-WT) and the T4SS-defficient mutant (Δ*dotA*) in hMDMs cultured in nutrient-limited media supplemented with either 10 mM glucose or 10 mM galactose. Data represent the mean ± SD the bacterial area (μm²) per infected cell over time of four replicates from one experiment, representative of two independent experiments with two different donors.

Minor comments:

- Line 41: The authors write “are required for”, but given their findings, it might be more accurate to phrase this as “have previously been described to be required for” or “have previously been described essential for”.

We agree. We will change it.

- Line 86: Is the referencing of Figure S1C correct or should it be S1A?

Yes, thank you, it is S1A, we will change it.

- Lines 119,120: Related to what is displayed in Figure 2G: Are these differences significant?

Glucose, galactose and lactate curves are significantly different compared to control ($p<0.001$ by one-way ANOVA of the area under the curve, AUC). We will add this analysis to the main text.

- Lines 126,127: What is the change for glycerol, and is the intracellular growth significantly higher compared to the control?

  6,2 ± 1.9% in glycerol vs. 2 ± 1% in control, $p<0.05$. We will add this comparison in the main text and Line 127.

- Figure 1E&F: Related to one of the major comments: Would it be possible to quantify this at timepoint 0 to ensure that the initial infection rates are the same across conditions?

  As said before, we will add the graphs of % of infected cells at t=0 as supplementary Figures S1G-K (Major Comment number 2 from this Reviewer)

- Figure 3E,F: Why does the sum of the curves not add up to 100% (especially in the beginning)? And related to that, why do both the percentage of cytosolic and vacuolar cells grow over time? Since this infection is performed with gentamycin present, re-infection should not be possible.

  The localization module of the SINA plasmid relies on transcriptional reporters, whose expression requires time for induction and detection. Therefore, at early time points, no infected cells are classified as vacuolar or cytoplasmic because the reporters have not yet been expressed (as described in *PLoS Pathog*. 2021;17(4):e1009550, PMID: 33930101).

  At later time points, a subset of cells harbor bacteria that do not express any of the reporters. These bacteria are considered dormant, representing about 10% of the population, as detailed in the same article. In addition, a small percentage of infected cells simultaneously contain both STvac and STcyt. Such cells are subclassified as harboring STcyt but also STvac. Consequently, the total proportion of infected cells carrying STvac and STcyt may also exceed 100%.

- Figure S1A: While significance testing is described in the legend, there are no indications of significance in the figure panels.

  The Reviewer is right, there is no significant changes between conditions, we will change the significance testing to ns=non-significant.

- Figure S1B: Due to the stark discrepancies between hMDMs and RAW264.7, it might make sense to plot them on two different y-axes. Furthermore, I would clarify the y-axis: In the legend, it seems as CFU counts are shown, while CFU/ml/t2 rather describes a change over time.

  We agree. However, we will maintain the scale of the Y-axis as it was required by Reviewer #1 to be consistent with Y-axis. We will change the legend to indicate that we plot CFU/ml/t2.

- Figure S1C: The prgH-mutant seems to outperform the wildtype in intracellular proliferation, while the double mutant underperforms compared to the ssaV-mutant. Could you please

discuss/explain how the prgH-deletion has seemingly opposite effects on intracellular proliferation, depending on whether it is introduced in a wildtype or ssaV-KO background?

> As T3SS-1 plays a role in inducing macrophage cell death via activation of the NLRC4 inflammasome, macrophages infected with bacteria carrying a functional T3SS-1 (such as WT), are more prone to undergo cell death at late time-points, which disrupts bacterial proliferation and reduces the proportion of infected cells. Thus, these dead cells stopped being considered in the analysis. Even if cell death of ST-WT-infected RAW macrophages remains below 5%, more Δ*prgH*-infected cells are considered in the analyses at late time-points, and ST-Δ*prgH* continue replicating (and growing in ST area).

- Figure S2A: As for the comments related to Figure 3, I am unsure how the sum of STvac and STcyt can deviate from 100. This is especially puzzling for the red curve (glycerol) at e.g. 3hpi, when the sum of the two clearly seems to be larger than 100.

> We answered to this in question before. At early time points, no infected cells are classified as vacuolar or cytoplasmic because the reporters have not yet been expressed. At later time points, a subset of cells harbor bacteria that do not express any of the reporters, which are considered dormant (10% of the population). Finally, a small percentage of infected cells simultaneously contain both STvac and STcyt, therefore the total proportion of infected cells carrying STvac and STcyt may also exceed 100%.

**Cross-commenting**
I agree in principle with the comments raised by Reviewer #1 - especially when it comes to the enhancement in significance if the authors assess the species specificity. Elucidating whether the growth enhancement is *Salmonella*-specific, occurs for other intracellular pathogens (*e.g. Shigella*, *Francisella*) or also for extracellular bacteria (e.g. *E. coli*, *Yersinia*), would definitely strengthen the study.

> As said before, for the revision we are going to perform the experiments suggested by Reviewer #1 of using E. coli K12 (ECK12) while culturing macrophages on different carbon sources (glucose, glycerol, galactose, fructose). And to satisfy this Reviewer's curiosity, we are going to perform experiments also with *L. pneumophila* and glycerol.

Reviewer #2 (Significance):

General assessment:

As the authors write in their discussion, the strength of this study is also it's limitation: Using single cell tracking in microscopy is a very elegant and powerful approach, yet conversely, it limits the scope of the study to *in vitro* approaches. While it enables assessment of bacterial pathogenicity and host-dependence on a single-cell level, it remains to be investigated whether the conclusion that the authors draw from their work will hold in more complex or physiologically relevant models.

We will discuss the implications of our results in more complex/physiological models in the Discussion of the revised manuscript.

A further small shortcoming of the study is the heavy focus on the bacterial aspect in this host-pathogen interaction. While the authors do link the proliferative potential of the intracellular bacteria to the metabolic status of the individual host cell, more could be done with respect to host responses in the varying media compositions, including investigating alterations to the cell cycle, induction of cell death, or the ability to activate inflammatory signaling.

We agree, and we are actively investigating how restricting macrophages to specific carbon sources impact other host responses, such as cytokine production. For the revised manuscript, we will add the results on the induction of cell death.

Nonetheless, this study is of large interest to the field and the systematic approach to addressing their hypotheses speaks to the scientific excellence of the investigators.

Thank you.

## 3. Description of the revisions that have already been incorporated in the transferred manuscript

N/A

## 4. Description of analyses that authors prefer not to carry out

N/A
