# Supplementary material for: Provision of preferred nutrients to macrophages promotes Salmonella intracellular replication without relying on Type III secretion systems
Source: PLoS Pathog. 2026 Jul 9;22(7):e1014348. doi: 10.1371/journal.ppat.1014348 (PMC13349091; doi:10.1371/journal.ppat.1014348)
Supplement: Attachment — Submitted filename: point-by-point-answers.pdf [file ppat.1014348.s007.pdf]

POINT-BY-POINT ANSWERS TO REVIEWERS

Reviewer #1

Evidence, reproducibility and clarity

In this manuscript, the authors investigate how host cell metabolic heterogeneity influences the intracellular replication of *Salmonella enterica* serovar Typhimurium. They use live-cell imaging of infected human primary macrophages to reveal that bacterial replication does not occur uniformly across infected cells. They demonstrate that supplementation with specific carbon sources-used by *Salmonella* during infection-promotes bacterial replication and increases the proportion of macrophages supporting intracellular growth. These effects are seen even in the absence of functional Type III Secretion Systems (T3SS), using a Δ*prgH*/Δ*ssaV* double mutant. The authors further suggest that this replication enhancement is not strictly dependent on host glycolytic activity but rather on the host cell's ability to import nutrients. Their findings imply that intracellular *Salmonella* can exploit host cell metabolism to grow, even without its canonical virulence secretion systems, under nutrient-favorable conditions.

Major Concern:

While the topic is potentially interesting, the novelty is not fully clear. The concept that nutrient availability impacts intracellular *Salmonella* replication, largely via T3SS2 function, has been addressed previously (e.g., Liss et al., 2017). The finding that added exogenous carbon sources can enhance bacterial growth is thus not unexpected. The key claim-that *Salmonella* can replicate intracellularly even in the absence of T3SS function-would be significantly strengthened by demonstrating whether this is specific to *Salmonella*, or whether similar effects are seen with non-intracellular organisms such as *E. coli* K-12. If the phenomenon is unique to *Salmonella*, this would suggest a pathogen-specific mechanism beyond general metabolic support.

As acknowledged by the Reviewer, the novelty and key claim of our work is that *Salmonella* can replicate intracellularly even in the absence of T3SS. This is clearly stated in the title of the manuscript. To experimentally sustain that claim, we showed evidence that providing macrophages with the preferred carbon sources used by *Salmonella* during infection, such as glycerol, bypass the requirement of both T3SS by *Salmonella* to grow, intravacuolarly, inside macrophages.

Regarding the key article mentioned by the Reviewer (Liss *et al.* 2017, ref 36 in the manuscript), we report three important novel insights: *i)* we show that *Salmonella* can replicate intracellularly in the SCV even in the absence of T3SS if certain carbon sources are provided; *ii)* we show the preference of *Salmonella* for certain carbon sources intracellularly such as glycerol and galactose (but not preferentially glucose); and *iii)* we have extended our observations to primary human macrophages in addition to RAW cells.

As requested by the Reviewer, we have performed experiments using *E. coli* K12 (ECK12). Briefly, we infected hMDMs with ST-Δ*prgH*Δ*ssaV* or ECK12-GFP, and the macrophages were cultured using different carbon sources (glucose, glycerol). We monitored intracellular bacterial growth, showing that ECK12 cannot grow inside macrophages when adding favorable carbon sources (glycerol) compared to *Salmonella* double mutant-infected cells. This indicates that this phenomenon is unique to *Salmonella* and cannot be reproduced in *E. coli*. We added this results as **new Figure S1B**. Lines 112-114 read now: *"The intracellular growth of a T3SS double mutant is specific as E. coli even when given preferred carbon sources cannot grow intracellularly in macrophages (**Figure S1B**), indicating that additional virulence strategies to the T3SSs are needed to replicate within macrophages when cultured in the presence of glycerol."*

Specific Comments:

1. Figure 1H: The effect shown here is not compelling due to inconsistent y-axis scaling. Panels 1B, 1C, and 1D should use a unified axis range with 1H to allow direct visual comparison of growth dynamics.

We have changed it as requested.

2. Figures 1B, 1C, 1G, 1H: The current presentation of individual growth traces makes it difficult to appreciate the population-level trend. A smoothed average line overlaid on these plots could better represent the average dynamics of replicative vs. non-replicative infections. Or alternatively the total fraction of cells that proliferate summarized as a segmented bar plot (possibly binned per time point).

We implemented the Reviewer's suggestion and now present the total fraction of infected cells harboring replicative bacteria as segmented bar plots for all carbon sources (**new Figures S1A, S1E**). In our pipeline, the replicative vs. non-replicative classification is assigned retrospectively by the algorithm once divergence in growth relative to the initial time points can be validated at later time. Accordingly, the labels "cell with replicative bacteria" and "cell with non-replicative bacteria" apply to the entire trajectory. This

retrospective assignment explains why some trajectories appear as “replicative” at t=0 in Figures 1B, G: the bacterium is not replicating at that instant but is subsequently confirmed, at later time-points, to do so. Because the classification is trajectory-wide rather than time-resolved, binning by time does not yield meaningful differences across time points, and a smoothed average line over single-cell traces would obscure the underlying bimodality of single-cell tracks. We therefore summarize the data as aggregated fractions over full trajectories in Figures S1A and S1E, as requested.

3. Figure 2G: This panel would benefit from including a comparable condition with the SPI-1/SPI-2 double mutant to aid interpretation. Additionally, the authors should explore whether this nutrient-supported replication is seen in non-phagocytic cells such as HeLa or Caco-2, which would help delineate whether the observed phenomenon is macrophage-specific.

The plot asked by the Reviewer is **Figure S1F.** As we are representing ST growth in macrophages supporting *Salmonella* replication, some of the conditions, such as lactate, cannot be shown in the infection conditions using the double SPI-1/SPI-2 mutant because there are no cells supporting bacterial replication, so there are no cells to plot. New **Figures S1A and S1E** also addresses the request of the Reviewer.

As suggested, we have performed the same experiments in HeLa cells to know whether the observed phenomenon is macrophage-specific (**new Figure S1G**). Our results indicate that the effect of glycerol is specific of human macrophages. Lines 151-153: *“Although the replication of the T3SS-deficient strain can also be observed in HeLa cells (Figure S1G), the higher effect of glycerol promoting the growth of the double mutant compared to glucose is specific of human macrophages”.*

4. Line 117: The sentence stating that the double mutant can undergo “exponential intracellular growth even in the absence of T3SS-dependent secretion” is an overstatement. The data suggest only a modest improvement in growth, restricted to a minority of infected cells. This claim should be revised accordingly, as should similar overstatements in the discussion (e.g., lines 203-204).

As requested by the reviewer, we have removed the term “exponential” at line 137 and have revised the sentence at line 124 and those in the discussion, lines 242-243: *“we demonstrated that providing macrophages with preferred nutrients allows a subpopulation of ST to replicate intracellularly without the need for a functional T3SS (T3SS1 and T3SS2)”*.

5. Line 162: The authors should clarify that glycerol had the strongest effect in primary macrophages, while multiple alternative carbon sources had notable effects primarily in RAW cells.

We thank the reviewer for this suggestion; we have added this clarification in the text. Lines 182-183: *"Thus, glycerol had the strongest effect in primary human macrophages, while multiple alternative carbon sources had notable effects in RAW cells."*

6. Lines 198-201: This relates to the major concern. The authors should assess whether the observed growth enhancement is unique to Salmonella by testing other bacteria not known for intracellular replication. This would clarify whether the effect is due to general nutrient-driven host cell permissivity or a pathogen-specific adaptation.

We have performed the suggested experiments with *E. coli* K12 showing that this phenomenon is unique to *Salmonella* (**new Figure S1B**), please see previous comment.

7. RAW 264.7 Observations: The modest intracellular growth of SPI-1/SPI-2 double mutants in RAW cells is consistent with prior observations in the field. The idea that nutrient availability explains this is noteworthy. The authors might consider whether differences in standard culture media (e.g., glucose concentration) influence these outcomes. This could have broader implications for reproducibility in infection models.

Thank you for the suggestion. To answer also a question from Reviewer #2, we have included a new supplementary Figure comparing no Glucose (0 mM), low Glucose (2 mM) and standard culture media Glucose levels (10 mM), showing that differences in Glucose levels in the culture media influence *Salmonella* intracellular growth in hMDMs and RAW macrophages (**new Figures S3E-F**). We have also updated the text, lines 233-235: *"Moreover, glucose supplementation showed a gradual effect on T3SS-independent intracellular replication of ST in RAW macrophages (**Figures S3E** and **S3F**), which might have broader implications for reproducibility in infection models."*

Significance

This manuscript highlights how host cell metabolism and nutrient availability can influence intracellular Salmonella replication. While the findings are intriguing, the current framing overstates their novelty and impact. Key revisions-such as comparative experiments with non-pathogenic bacteria and non-phagocytic cells, consistent figure scaling, and more measured language-would improve the clarity and significance of the work. If the authors can show

Salmonella-specific mechanisms at play, the study could offer important insights into host-pathogen metabolic interactions.

We thank the reviewer for the constructive feedback. We have performed the requested new experiments and revised the text to remove overstatements. We believe, these changes strengthen the manuscript and provide clearer insight into how host–pathogen metabolic interactions shape *Salmonella* virulence. At single-cell resolution, we show that only a subset of infected macrophages supports intracellular replication. This subset expands when macrophages are supplemented with relevant carbon sources. Remarkably, even a Δ*prgH*/Δ*ssaV* double mutant replicates in a fraction of cells under nutrient-favorable conditions.

Together, our results support two conclusions. First, the metabolic state of host cells is a primary determinant of intracellular bacterial growth. When nutrient access is favorable, T3SSs are not strictly required for replication. Second, T3SS effectors remain important in physiological settings because they remodel host metabolism and endosomal trafficking to generate nutrient-permissive niches. When that metabolic state is achieved by other means, T3SS dependence can be bypassed. As discussed with the Editor, we now state these points explicitly, but cautiously, in the Abstract, lines 26-29: "*In summary, our findings suggest that the dependence of Salmonella on its T3SSs for intracellular replication can be bypassed when host cells provide optimal access to key nutrients. This underscores the role of host cell metabolism in shaping intracellular bacterial replication and highlights the crucial functions of T3SS effectors in remodelling macrophage metabolism and endosomal trafficking to establish nutrient-permissive niches. When equivalent metabolic conditions are achieved by other means, dependence on T3SSs becomes dispensable*". We believe these additions address the concern and improve the clarity and balance of our conclusions.

Reviewer #2

Evidence, reproducibility and clarity

Summary:

In their study titled "Provision of Preferred Nutrients to Macrophages Enables Salmonella to Replicate Intracellularly Without Relying on Type III Secretion Systems", Dr. Garcia-Rodriguez et al. describe the influence of the host cell metabolism on the intracellular proliferation potential of *Salmonella* during infection. The authors investigate whether the supplementation of the media with different carbon sources has an impact on the intracellular lifestyle of Salmonella. By using single cell tracking in live-cell microscopy, including the use of different reporter strains, they describe that glycerol benefits *Salmonella*'s ability to grow within its vacuolar niche, in part, interestingly, in a Type-3-Secretion System independent manner.

They furthermore highlight the dependence on host background for this observation by showing that effects differ between cells of varying metabolic activity. Throughout their study, they use cutting-edge methodologies, as well as *Salmonella* strains that could be of versatile use in other investigations. This work, while limited to in vitro models for now, has implications for the better understanding of how pathogens and their host are intertwined. This, in turn, has significance for the development of new anti-infective strategies further down the line. I therefore believe that it should be disseminated to the research community. The following comments summarize ideas how the quality of the study could be improved:

Major comments:

1. *Salmonella*, especially when cultured to activate the SPI-1 T3SS, introduce rapid cell death in their host - most commonly through activation of the NLRC4 inflammasome and downstream pyroptotic signaling. The authors don't describe the effect of the infection in differently supplemented media on host cell death, yet it would be important to elucidate whether this cellular response is also altered.

> We have performed these experiments and tracked host cell death by measuring Annexin V levels in single cells during infection in supplemented media with different carbon sources. Our results clearly indicate that cell death is not affected by supplementation with different carbon sources. We have included these results in the revised version of the manuscript and main text **(new Figure S2)** Lines 153-154: "*Supplementation of macrophages with these different carbon sources did not noticeably affect initial bacterial infection (Figure S1H to S1L) nor host cell death during infection (Figure S2)*".

2. The aspect of partially T3SS-independent growth enhancement by glycerol (and depending on the host background glucose) is most curious. The authors quantify this by determining the percentage of cells containing proliferating Salmonella and by tracking individual cells over the time course of the infection. I am missing a general statement on whether the initial infection rate (i.e. timepoint 0) is comparable across conditions and mutants, and whether possible discrepancies in the infection rate could have downstream effects on the statements and claims made in the manuscript. This is, to my mind, also important for the quantification of cytosolic and vacuolar bacteria. There, the authors always speak in "percent of infected cells", so it is relevant whether the number of infected cells varies among conditions (see e.g. Figure 3).

We thank the reviewer for this comment. The initial infection rate at t=0 significantly differs between WT and mutants in RAW 264.7 macrophages, and carbon source supplementation has no effect. However, as we only analyze infected cells, this does not affect the final results. In any case, we have added the graphs of % of infected cells at t=0 as **new supplementary Figures S1H-L** and updated the text, Lines 153-154: "*Supplementation of macrophages with these different carbon sources did not noticeably affect initial bacterial infection (Figure S1H to S1L)*"

3. The authors use a concentration of 10mM for all supplemented alternative carbon sources. It would be useful to discuss the rationale behind this approach, including whether all chemicals have the same ability to be taken up by the cell. A concentration series (at least for some of the tested compounds) may be beneficial to bolster the conclusions that the authors make.

We use 10 mM as this is the concentration of Glucose in standard culture media. By using 10 mM for all the different carbon sources, we can thus compare them keeping concentration constant (10 mM). As this Reviewer suggested, we have included a new supplementary Figure comparing no Glucose (0 mM), low Glucose (2 mM) and standard culture media Glucose levels (10 mM), showing that the concentration of glucose has a gradual effect in supporting the replication of the T3SS-deficient strain in RAW macrophages (**new Figures S3E-F**). Indeed, this question was also asked by Reviewer #1. We have also updated the text, lines 233-235: *"Moreover, glucose supplementation showed a gradual effect on T3SS-independent intracellular replication of ST in RAW macrophages (**Figures S3E** and **S3F**), which might have broader implications for reproducibility in infection models."*

4. I think it would strengthen the study, if the authors used host cell mutants in certain metabolite transporters, or alternatively Salmonella mutants that are deficient in uptake or metabolism of some of the compounds used in this study. This point is alluded to in the discussion, and I believe if the authors could show that in certain host mutant backgrounds the impact of supplementation with alternative carbon sources can be reversed, it would immensely bolster the strength of the claims.

Following Reviewer's suggestion, we generated ST metabolic mutants unable to metabolize glycerol, galactose or fructose. As can be seen in **new Figure 4J**, during infection, the supplementations with glycerol/galactose does not boost *Salmonella* replication in metabolic mutants as in WT conditions, demonstrating that supplemented carbon sources indeed arrive to bacteria within the SCV and are used by intracellular *Salmonella* to grow.

These results are now presented in the main text in Lines 223-233: *"To determine whether the benefit of supplementation requires bacterial catabolism of the provided sugars, we infected hMDMs with ST-WT or isogenic metabolic mutants unable to metabolize galactose (ΔgalK), fructose (ΔfruK), or glycerol (ΔglpK) and quantified the percentage of infected cells containing replicative bacteria across matched supplements (10 mM glucose, galactose, glycerol, or fructose). As predicted, the matched defects abrogated the advantage conferred by the corresponding supplement, and ST-ΔgalK failed to increase replication with galactose, ST-ΔglpK failed with glycerol, and ST-ΔfruK showed no improvement with fructose, whereas each mutant recovered when provided with an alternative carbon source it could metabolize, such as glycerol, mirroring ST-WT responses (**Figure 4J**). These results demonstrate that supplemented carbon sources indeed arrive to bacteria within the SCV and are used by intracellular Salmonella to grow. Thus, nutrient-driven increase in intracellular replication depends on the bacterial capacity to catabolize the available metabolite, not merely on host sugar uptake or overall glycolytic activity."*

5. I think it would be useful to include the meaning of this work for other intracellular pathogens in the discussion section: Do the authors believe that this phenotype is *Salmonella*-specific? If the pathogens are at hand, it might be interesting to infect with other intracellular bacteria, such as Shigella or Francisella to investigate if the boosting of growth by glycerol also holds true for these.

We have performed experiments with *Legionella pneumophila* and galactose (see figure below, **R1**), showing that this carbon source is in this case specific of *Salmonella* (as shown in Figure 4F in the manuscript). We think that results using *Legionella* might be out of the

focus of this article and would constitute themselves part of a new article, as both pathogens have a very different, non-comparable intracellular metabolism. Requested by Reviewer #1, we have also performed experiments using *E. coli* K12 (ECK12). Briefly, we infected hMDMs with ST-Δ*prgH*Δ*ssaV* or ECK12, while culturing macrophages on different carbon sources (glucose, glycerol). We monitored intracellular bacterial growth, showing that ECK12 cannot grow inside macrophages under favorable carbon sources (glycerol) as is seen in double mutant-infected cells. This indicates that this phenomenon is unique to *Salmonella* and cannot be reproduced in *E. coli.* We added this results as **new Figure S1B**. Lines 112-114 now read: *"The intracellular growth of a T3SS double mutant is specific as E. coli even when given preferred carbon sources cannot grow intracellularly in macrophages (**Figure S1B**), indicating that additional virulence strategies to the T3SSs are needed to replicate within macrophages when cultured in the presence of glycerol."*

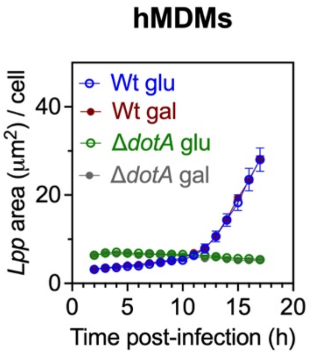

Figure R1. Only for the Reviewer. Galactose supplementation does not enhance intracellular growth of *Legionella pneumophila* in hMDMs. Intracellular growth dynamics of *Legionella pneumophila* strain Paris wild-type strain (*Lpp*-WT) and the T4SS-defficient mutant (Δ*dotA*) in hMDMs cultured in nutrient-limited media supplemented with either 10 mM glucose or 10 mM galactose. Data represent the mean ± SD the bacterial area (µm²) per infected cell over time of four replicates from one experiment, representative of two independent experiments with two different donors.

Minor comments:

- Line 41: The authors write "are required for", but given their findings, it might be more accurate to phrase this as "have previously been described to be required for" or "have previously been described essential for".

We agree. We changed it (Line 59)

- Line 86: Is the referencing of Figure S1C correct or should it be S1A?

Sorry for the mistake, was changed (Line 107).

- Lines 119,120: Related to what is displayed in Figure 2G: Are these differences significant?

Glucose, galactose and lactate curves are significantly different compared to control ($p<0.001$ by one-way ANOVA of the area under the curve, AUC). We added this analysis to the main text (lines 141-142)

- Lines 126,127: What is the change for glycerol, and is the intracellular growth significantly higher compared to the control?

6,2 ± 1.9% in glycerol vs. 2 ± 1% in control, $p<0.05$. We added this comparison in the main text, lines 148-149.

- Figure 1E&F: Related to one of the major comments: Would it be possible to quantify this at timepoint 0 to ensure that the initial infection rates are the same across conditions?

We added the graphs of % of infected cells at t=0 as **new supplementary Figures S1H to S1L.** Lines 153-154 read now: "*Supplementation of macrophages with these different carbon sources did not affect initial bacterial infection (Figure S1H to S1L) nor host cell death during infection (Figure S2)".*

- Figure 3E,F: Why does the sum of the curves not add up to 100% (especially in the beginning)? And related to that, why do both the percentage of cytosolic and vacuolar cells grow over time? Since this infection is performed with gentamycin present, re-infection should not be possible.

The localization module of the SINA plasmid relies on transcriptional reporters, whose expression requires time for induction and detection. Therefore, at early time points, no infected cells are classified as vacuolar or cytoplasmic because the reporters have not yet expressed (as described in PLoS Pathog. 2021;17(4):e1009550, PMID: 33930101).

At later time points, a subset of cells harbors bacteria that do not express any of the reporters. These bacteria are considered dormant, representing about 10% of the population, as detailed in the same article. In addition, a small percentage of infected cells contain both, STvac and STcyt, which are then counted double thus the total proportion of infected cells may also exceed 100%.

- Figure S1A: While significance testing is described in the legend, there are no indications of significance in the figure panels.

Sorry for this oversight, there are no significant changes between conditions. We have removed the significance testing from the legend.

- Figure S1B: Due to the stark discrepancies between hMDMs and RAW264.7, it might make sense to plot them on two different y-axes. Furthermore, I would clarify the y-axis: In the legend, it seems as CFU counts are shown, while CFU/ml/t2 rather describes a change over time.

We agree. However, we have maintained the scale of the Y-axis as it was required by Reviewer #1 to be consistent with Y-axis. We have changed the legend to indicate that we plot CFU/ml/t2.

- Figure S1C: The prgH-mutant seems to outperform the wildtype in intracellular proliferation, while the double mutant underperforms compared to the ssaV-mutant. Could you please discuss/explain how the prgH-deletion has seemingly opposite effects on intracellular proliferation, depending on whether it is introduced in a wildtype or ssaV-KO background?

As T3SS-1 plays a role in inducing macrophage cell death via activation of the NLRC4 inflammasome, macrophages infected with bacteria carrying a functional T3SS-1 (WT and Δ*ssaV*), are more prone to undergo cell death at late time-points, which disrupts bacterial proliferation and reduces the proportion of infected cells. Thus, these dead cells are not being considered in the analysis. Even if cell death of ST-WT-infected RAW macrophages remains below 5%, more Δ*prgH*-infected cells are considered in the analyses at late time-points, and ST-Δ*prgH* continue replicating (and growing in ST area).

- Figure S2A: As for the comments related to Figure 3, I am unsure how the sum of STvac and STcyt can deviate from 100. This is especially puzzling for the red curve (glycerol) at e.g. 3hpi, when the sum of the two clearly seems to be larger than 100.

Please find our full explanation under the comments related to Figure 3. At early time points, no infected cells are classified as vacuolar or cytoplasmic because the reporters have not yet been expressed. At later time points, a subset of cells harbors bacteria that do not express any of the reporters, which are considered dormant (10% of the population). Finally, a small percentage of infected cells contain both STvac and STcyt which are then counted double, therefore the total proportion of infected cells carrying STvac and STcyt may also exceed 100%.

Cross-commenting

I agree in principle with the comments raised by Reviewer #1 - especially when it comes to the enhancement in significance if the authors assess the species specificity. Elucidating whether the growth enhancement is Salmonella-specific, occurs for other intracellular pathogens (e.g. *Shigella*, *Francisella*) or also for extracellular bacteria (e.g. *E. coli*, *Yersinia*), would definitely strengthen the study.

We performed the experiments suggested by Reviewer #1 using E. coli K12 (ECK12) while culturing macrophages on different carbon sources (glucose and glycerol, **new Figure**

S1B). And, we have shown results of *L. pneumophila* and galactose for the reviewer (**Figure R1**).

Significance

General assessment:

As the authors write in their discussion, the strength of this study is also it's limitation: Using single cell tracking in microscopy is a very elegant and powerful approach, yet conversely, it limits the scope of the study to in vitro approaches. While it enables assessment of bacterial pathogenicity and host-dependence on a single-cell level, it remains to be investigated whether the conclusion that the authors draw from their work will hold in more complex or physiologically relevant models.

We have discussed the implications of our results in more complex/physiological models in the Discussion, lines 294-299: *"Importantly, previous in vivo studies directly support our central conclusion. Grant et al. used multicolor fluorescence microscopy in infected murine tissues to show that SPI-2-deficient S. Typhimurium can nonetheless replicate to high intracellular densities within phagocytes, indicating that the SPI-2 T3SS is dispensable for intramacrophage replication in vivo (39). Our single-cell, metabolically focused data provide a mechanistic explanation consistent with those observations by proposing that sufficient access to preferred host nutrients can enable T3SS-independent growth within macrophages."*

A further small shortcoming of the study is the heavy focus on the bacterial aspect in this host-pathogen interaction. While the authors do link the proliferative potential of the intracellular bacteria to the metabolic status of the individual host cell, more could be done with respect to host responses in the varying media compositions, including investigating alterations to the cell cycle, induction of cell death, or the ability to activate inflammatory signaling.

We agree, and we are actively investigating how restricting macrophages to specific carbon sources impact other host responses, such as cytokine production. In the revised manuscript, we have added the results on the induction of cell death **(new Figure S2).**

Nonetheless, this study is of large interest to the field and the systematic approach to addressing their hypotheses speaks to the scientific excellence of the investigators.

We thank the reviewers for their constructive feedback and hope the incorporated changes strengthen the quality of the work and make it suitable for publication.
