# Supplementary material for: Provision of preferred nutrients to macrophages promotes Salmonella intracellular replication without relying on Type III secretion systems
Source: PLoS Pathog. 2026 Jul 9;22(7):e1014348. doi: 10.1371/journal.ppat.1014348 (PMC13349091; doi:10.1371/journal.ppat.1014348)
Supplement: Attachment — Submitted filename: point-by-point-answers_auresp_2.pdf [file ppat.1014348.s008.pdf]

POINT-BY-POINT ANSWERS TO REVIEWERS

Reviewer #1

In this study, the authors explore how variability in host cell metabolism shapes the intracellular growth of *Salmonella enterica* serovar Typhimurium. Using live-cell imaging in infected human primary macrophages, they show that bacterial replication is unevenly distributed, occurring only in a subset of infected cells. They find that adding particular carbon sources—known to be utilized by *Salmonella* during infection—enhances bacterial proliferation and increases the fraction of macrophages that permit intracellular replication. Notably, this stimulatory effect is also observed in a Δ*prgH*/Δ*ssaV* double mutant lacking functional Type III Secretion Systems (T3SS). The authors propose that the boost in replication is not primarily driven by host glycolysis itself, but instead depends on the host cell's capacity to take up nutrients. Overall, the data suggest that under nutrient-rich conditions, intracellular *Salmonella* can capitalize on host metabolic resources to replicate, even in the absence of its classical virulence-associated secretion systems.

1. This reviewer finds the title highly misleading, in the sense it implies the absence of T3SSs can be fully compensated by the right nutrient supplementation. The authors demonstrate only partial restoration of intracellular replication with nutrient supplementation, and only a fraction of that compared to T3SS-expressing STm. Unless the authors can demonstrate complete replication competence comparable to WT STm with nutrient supplementation, the authors should tone down the phrasing of the title, as was acknowledged and done by the authors based on the former reviewers' requests. To argue that the T3SS is indispensable for intracellular STm growth is not supported by the evidence presented in the manuscript.

> To avoid any potential overinterpretation, we agree to revise the title by replacing "enables" with the more moderate term "promotes", which might more accurately reflect the observed phenotype. Now the title reads: ***"Provision of Preferred Nutrients to Macrophages Promotes Salmonella Intracellular Replication Without Relying on Type III Secretion Systems"***

2. Authors should exercise more caution in their interpretation and phrasing. For example, this reviewer strongly recommends to rephrase wording in the abstract and throughout, such as L29: 'When equivalent metabolic conditions are achieved by other means, dependence on T3SSs becomes dispensable' to 'dependence on T3SSs for intracellular replication can be partially compensated *in vitro*'. STm T3SS mutants (SPI-1 and/or SPI-2) are highly attenuated for virulence in in vitro and in animal models. This reviewer remains sceptical as to whether

any amount of nutrient supplementation in any *in vitro* or *in vivo* model (while the latter are admittedly are challenging experiments), is sufficient to fully compensate for the absence of one or both SPI-1/2 T3SSs. I therefore encourage the authors to exercise more caution in suggesting that the T3SS is dispensable, as long as the right nutrients are provided. At best what the authors show is that it partially restores intracellular replication of a STm-T3SS-deficient mutant *in vitro*. There is no evidence yet to demonstrate this effect is meaningful for virulence/pathogenesis in an infection scenario.

We never claimed a full compensation of virulence by nutrient supplementation. To avoid potential overinterpretation of our results by readers, we have revised these sentences as suggested by the Reviewer:

Line 25: *"In summary, our findings suggest that the dependence Salmonella on its T3SSs for intracellular replication can be compensated, at least partially, when host cells provide optimal access to key nutrients"*

Line 29: *"When equivalent metabolic conditions are achieved by other means in vitro, dependence on T3SSs for intracellular replication can be partially compensated in macrophages"*

3. Figure S1G seems to be missing a control, only glycerol and glucose conditions are shown. Also, the experiment in HeLa cells should not be performed with a double mutant, rather a SPI-2 mutant only, as a SPI-1 deficient STm will not allow invasion into HeLa cells at all, an thus have no infected cells to measure. Thus, such an experiment should be performed with a single SPI-2 mutant.

We thank the reviewer for raising these points. Regarding the missing control condition in Figure S1G, we were unable to include HeLa cells cultured in nutrient-limited medium without any carbon source because HeLa cells display very poor viability under these conditions, with extensive cell death occurring within the first hours of the experiment, precluding any meaningful analysis of intracellular bacterial dynamics. For this reason, the comparison shown in Figure S1G is restricted to glycerol versus glucose supplementation, and the conclusion we draw from this panel is specifically that the enhanced effect of glycerol over glucose on intracellular replication, which is clearly observed in human primary macrophages (Figure 1E and 1F), is not recapitulated in HeLa cells, where both carbon sources support similar levels of bacterial replication. We have revised the text to make this specific claim clearer and to avoid broader statements about macrophage specificity that are not fully supported by the current dataset.

Line 154 now reads: *"Although some replication of the T3SS-deficient strain can also be observed in HeLa cells, the preferential effect of glycerol over glucose in promoting intracellular growth of the double mutant was only observed in human primary macrophages (Figure S1G)."*.

Regarding the use of the ST-Δ*prgH*/Δ*ssaV* double mutant in HeLa cells, we appreciate the reviewer's concern. While it is true that SPI-1 deficiency dramatically reduces invasion of epithelial cells, the double mutant does infect a small but measurable fraction of HeLa cells, particularly at the higher MOI used in our experiments (MOI=50). Importantly, as indicated in the figure axes, all our analyses are performed exclusively on infected cells. This means that only cells containing intracellular bacteria are included in the quantification of replication dynamics. We show for the Reviewer the percentage of infected HeLa cells for both ST-WT and the ST-Δ*prgH*/Δ*ssaV* strain to document the lower but non-zero infection rate of the double mutant (Figure R1, only for the reviewer). We chose to use the double mutant in HeLa cells to maintain consistency with the macrophage experiments, enabling direct comparison of the same bacterial strain across different host cell types.

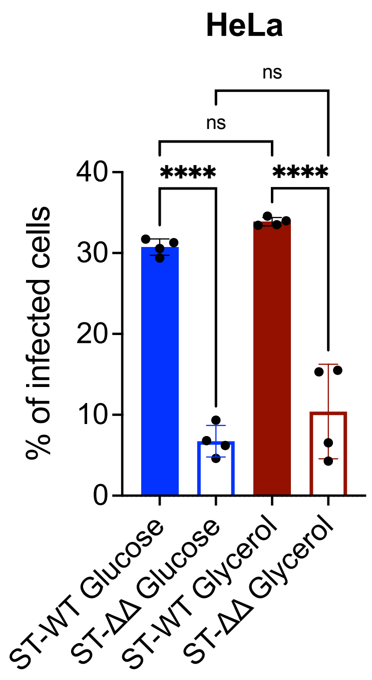

Figure R1. Related to Figure S1G. Percentage of HeLa cells infected by ST-WT or ST-ΔΔ strains when cultured in nutrient-limited medium supplemented with 10 mM glucose or 10 mM glycerol (****=p<0.0001, ordinary one-way ANOVA).

Minor issues

4. Figure 1. B, C, G ,H. The number of infected cells analysed in B, C, G and H need to be similar. Otherwise the traces become misleading. Currently C shows 187 single cell trajectories, whereas H, stands at 332. As these two conditions should be compared directly, it is important to have similar cell numbers. The authors could perform random sampling for each of the groups with a unified total number of cells per group. This will enable fairer comparison between the four groups and indication of the effect size the authors are observe compared to WT conditions, and double mutant +/- glycerol. The authors should also overlay a smoothed average for each 'replicating(red)' and 'non-replicating(blue)' population to better illustrate the population averages of these two subpopulations of infected cells over time.

We prepared a script to perform random subsampling, but each iteration of the random sampler produced different graphs, which is expected when removing a substantial fraction of the data. For instance, to display only 187 random tracks in Figure 1H (332 total tracks), as suggested by the Reviewer, 145 tracks (43% of the data) would need to be discarded. Each run of the subsampling procedure yielded a visually different result, raising the question of which specific random subset should be selected for publication, an arbitrary choice that we felt would undermine, rather than improve, the transparency of the data representation. We therefore opted for an alternative approach that addresses the Reviewer's underlying concern (the difficulty of visually comparing panels with different numbers of trajectories) but without discarding data. We now overlay the median trajectories for the replicating (red) and non-replicating (blue) subpopulations as thicker, solid lines on all single-cell tracking panels, as suggested by the Reviewer. These summary lines allow direct visual comparison of population-level dynamics across panels regardless of the total number of individual trajectories displayed. We applied this new design to Figures 1B, 1C, 1G, 1H, and 2E, 2F, and updated the corresponding figure legends accordingly.

5. Figure 1. F. The Y-axis in Figure 1F should be set to 100%, as it is on 1E. This is important for readers to gauge the size of the effects observed relative to WT conditions.

Done. We introduced a break in the y-axis to allow visualization of values in different ranges and alerted of the break in the Figure 1F legend.

6. Figure S3F x- axis should be made to 100% for a fairer comparison to Figure S2E

Done, now S3E and S3F have same y-axis 0-100%

7. The authors claim it is the metabolic niche of the host that dictates T3SS-independent intracellular proliferation. However, as the nutrient supplementations being tested are added exogenously in tissue culture media, the effect of the carbon source is via supplemented into the media, and since macrophages are constantly sampling and engulfing nutrients, the hosts' role in this process is probably more passive than what is suggested by the authors. Where the host is probably doing nothing more than passing the nutrients to STm via active and continuous engulfment and internalisation of extracellular media components, rather than the nutrients STm is exposed to in the SCV being the result of any direct and active shaping/modulation by the host per se. This is an important distinction, as it relates to the question as to whether the effect observed by the authors is a phenomenon that is only observable in in tissue culture experiments. An observation that this phenomenon is also

observed in non-phagocytic cells (e.g. HeLa) would argue in favor of the authors suggestion that the host plays a more active role, whereas if this phenomenon is only observed in phagocytic cells it could be a more passive effect of STm and media components being brought into close proximity within the endosomal compartment of infected cells. This relates to experiments suggested by former previous reviewer 1 and the suggested experiment above with the single SPI-2 mutant (minor point 5).

We thank the reviewer for this thought-provoking comment, which raises an important conceptual distinction between passive nutrient delivery via phagocytic uptake and active metabolic shaping by the host cell. We respectfully argue that several lines of evidence in our manuscript, together with recent literature (Fang et al, bioRxiv doi: 10.1101/2025.06.10.658782), support an active role of the host cell rather than a mere passive transfer of extracellular nutrients to the SCV.

First, if the mechanism were simply passive engulfment of medium components, macrophages would indiscriminately internalize whatever is present, and intracellular bacteria would be expected to phenocopy the replication patterns observed under axenic conditions, where supplementation with glucose, glycerol, galactose, or fructose yields similar growth levels (Figure R2, for the Reviewer). However, our results reveal clear carbon source selectivity: glycerol and galactose enhance intracellular growth relative to glucose (Figure 1D,E, 4E,F) whereas fructose, despite being present at identical concentrations, does not (Figure 4E,F). Moreover, the metabolic mutant experiments (Figure 4J) demonstrate that the supplemented carbon sources must be catabolized by the bacteria themselves, confirming that the nutrients do reach the SCV but that their effect depends on specific bacterial metabolic capacities, not on bulk delivery.

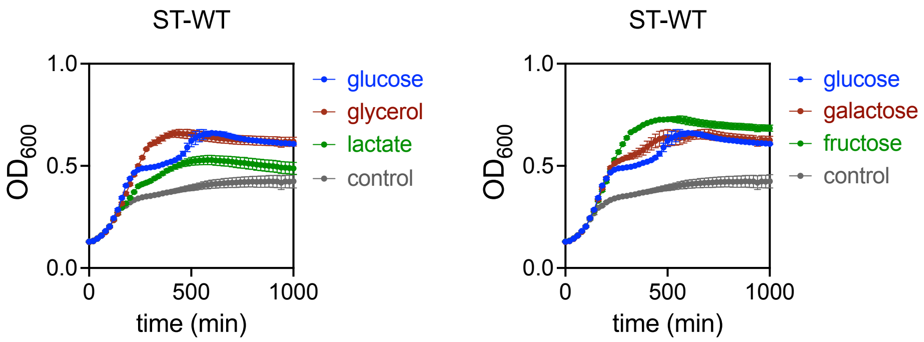

Figure R2. Monitoring of ST growth under axenic conditions in macrophage culture media. ST-WT was cultured in macrophage nutrient-limited (RPMI-based) medium supplemented with 10 mM of the indicated carbon sources. Optical density at 600 nm (OD_{600}) was measured every 15 min for 18 h.

Second, and critically, our single-cell analyses of glucose uptake (Figure 4H and 4I) show that among macrophages exposed to the same extracellular glucose concentration, only those with higher glucose uptake, which is an active, transporter-mediated process, subsequently supported bacterial replication. If nutrient delivery to the SCV were a passive consequence of phagocytic sampling, one would not expect such heterogeneity among cells bathed in identical media. This is consistent with recent findings showing that glucose uptake in macrophages during *Salmonella* infection is mediated by active upregulation of the glucose transporter GLUT1 (Fang et al, bioRxiv doi: 10.1101/2025.06.10.658782), rather than by passive internalization of extracellular fluid.

Third, the reviewer suggests that observation of this phenomenon in non-phagocytic cells would argue for an active host role. Indeed, as shown in Figure S1G, the T3SS-deficient double mutant can also replicate in HeLa cells, which are non-phagocytic and do not constitutively sample and engulf extracellular media. Please note that in Figure S1G we plot % of cells with replicative bacteria, thus this figure shows that WT and double mutant replicate in HeLa cells. This observation argues against a model based purely on phagocytic uptake of media components and instead supports a model in which nutrient availability within the infected cell, regardless of the route of nutrient entry, is the key determinant of intracellular bacterial growth. Furthermore, the fact that highly glycolytic cell types (RAW macrophages and HeLa cells) are more permissive for T3SS-independent replication than primary human macrophages (Figure 2D) is consistent with the metabolic activity of the host cell, rather than its phagocytic capacity, being the relevant variable.

We acknowledge that our *in vitro* supplementation approach cannot fully recapitulate the complexity of nutrient availability *in vivo* in the Discussion by explicitly addressing this limitation (Lines 302-306). Nevertheless, we believe the weight of evidence supports a model in which the metabolic state of the host cell actively shapes the intracellular nutrient environment, rather than serving as a passive conduit for extracellular nutrients.

Reviewer #2

During infection *Salmonella* resides within a host membrane-bound compartment called the *Salmonella*-containing vacuole (SCV). The SCV is a restrictive environment for *Salmonella* growth. One of the roles of the SPI-2 T3SS is to modify the SCV to enable intracellular replication. Here the authors show that intracellular replication is enhanced by the availability of carbon sources in the host cell media, and this is dependent on the ability of *Salmonella* to metabolise these carbon sources. Really interestingly, they show that providing host cells with supplementary carbon sources can even support the growth of T3SS mutants in a subset of cells. However, the authors need to take care (i) not to overstate these findings and (ii) to only make conclusions that are backed up by statistically significant data.

1. Effectors of the SPI-2 T3SS have multiple roles during systemic infection, including disrupting the host immune response. This should be made clear. Carbon source supplementation does not restore intracellular replication of T3SS mutants to the level of wt *Salmonella*. Therefore, suggestions that nutrient can "override the requirement of the T3SS", or that the "primary purposes of T3SS effector proteins is to overcome host-imposed nutrient restriction within macrophage vacuoles" should be toned down, including in lines 41, 264, 247, line 288 and line 306. It would be interesting to see if the replication defect of the SseFG deletion can be rescued by nutrient availability.

We have toned down our claims, as requested. We also acknowledge the Reviewer's important point that SPI-2 T3SS effectors have multiple roles during systemic infection beyond nutrient acquisition, including modulation of the host immune response and maintenance of the vacuolar replication niche. We have revised the relevant sections to reflect this broader view of T3SS effector functions. The specific changes are as follows:

Line 42 now reads: "*This indicates that one <u>important</u> function of these virulence factors, <u>among others</u>, is to secure access to nutrients.*"

Line 258: "*[…] under nutrient-rich conditions, Salmonella mutants deficient in both T3SS1 and T3SS2 (ST-ΔprgH/ΔssaV mutant strain) can replicate within <u>a subset of</u> macrophages, indicating that access to nutrients alone can partially substitute for T3SS-mediated virulence.*"

Line 273: "*<u>Among the multiple functions attributed to T3SS effectors, including maintenance of the vacuolar niche, immunomodulation, and remodeling of endosomal trafficking, nutrient acquisition appears to have a key role.</u> This suggests that one of the*

important functions of T3SS effector proteins is to overcome host-imposed nutrient restriction within macrophage vacuoles."

Line 299 now reads: "*Overall, our results support a model where the nutritional state of the macrophage can partially compensate the requirement for T3SS, highlighting the role of host metabolism in controlling intracellular infections*"

Line 317 now reads: "*In summary, our work revealed nutrient availability as a ~~critical~~ determinant that can circumvent, at least partially, the necessity of T3SS-mediated virulence of Salmonella.*"

Regarding the suggestion to test whether nutrient availability can rescue the replication defect of an SseF/G deletion mutant, we appreciate the Reviewer's interest. However, SseF and SseG are individual effectors secreted by the SPI-2 T3SS, and our ST-Δ*ssaV* mutant, which is deficient in the T3SS2 structural apparatus, already abolishes secretion of all SPI-2 effectors, including SseF and SseG. Since we demonstrate that nutrient supplementation can partially restore replication of this upstream mutant (which lacks all SPI-2 effector secretion), the prediction would be that the same or a greater rescue would be observed for a downstream single-effector deletion such as Δ*sseF/G*. We therefore believe that testing individual effector deletions, while interesting for future mechanistic dissection of specific effector contributions to nutrient access, would not add substantially to the conclusions of the present study.

2. The authors stress this as a macrophage-specific response. In response to previous reviewer #1 they have checked the phenotype in HeLa cells. Although this data in Fig S1G shows no difference between cells supplemented with glycerol or glucose, it doesn't include the control grown in nutrient limited media. Data from HeLa cells on the intracellular growth dynamics of replicative bacteria is also absent. It is therefore impossible to judge whether supplementation of HeLa cells with carbon sources affects *Salmonella* replication. Without this data, the authors should remove arguments that this is a macrophage specific effect. Additionally, *Salmonella* relies on the SPI-1 T3SS for infection of epithelial cell lines. It is therefore likely that the double T3SS mutant has very low infection rates in HeLa cells. It may therefore be better to use ΔssaV mutants for these experiments.

We thank the reviewer for this comment, which aligns with a similar point raised by Reviewer #1 (point 3). As detailed in our response to that comment, we were unable to include a nutrient-limited medium control for HeLa cells because these cells display very poor viability in the absence of any carbon source, with extensive cell death occurring within

the first hours of the experiment, which precludes meaningful analysis of intracellular bacterial dynamics. We agree with the Reviewer that, without this control, we cannot draw firm conclusions about whether carbon source supplementation broadly affects *Salmonella* replication in HeLa cells. We have therefore revised the text to remove broader claims of macrophage specificity. The conclusion we now draw from Figure S1G is narrower and specifically supported by the available data: that the preferential effect of glycerol over glucose in promoting intracellular growth of the T3SS-deficient strain (clearly observed in human primary macrophages) is not recapitulated in HeLa cells. Line 154 now reads: *"Although some replication of the T3SS-deficient strain can also be observed in HeLa cells, the preferential effect of glycerol over glucose in promoting intracellular growth of the double mutant was only observed in human primary macrophages (Figure S1G)."*

Regarding the use of the ST-Δ*prgH*/Δ*ssaV* double mutant rather than a single Δ*ssaV* mutant in HeLa cells, while SPI-1 deficiency does dramatically reduce invasion of epithelial cells, the double mutant nonetheless infects a small but measurable fraction of HeLa cells at the MOI used in our experiments (MOI=50). All analyses are performed exclusively on infected cells, so the low invasion rate does not bias the quantification of intracellular replication. We provide the percentage of infected HeLa cells for both ST-WT and the double mutant to document this (Figure R1, provided for the reviewer). We chose to maintain the double mutant for consistency with the macrophage experiments, enabling direct comparison of the same bacterial strain across host cell types. Using a different mutant (Δ*ssaV*) exclusively for HeLa experiments would introduce a confounding variable, as SPI-1 effectors such as SopE2 have been shown to actively reprogram macrophage metabolism (Jiang et al., Nature Communications 2021), and their presence in a single Δ*ssaV* mutant could independently influence the metabolic phenotypes under investigation.

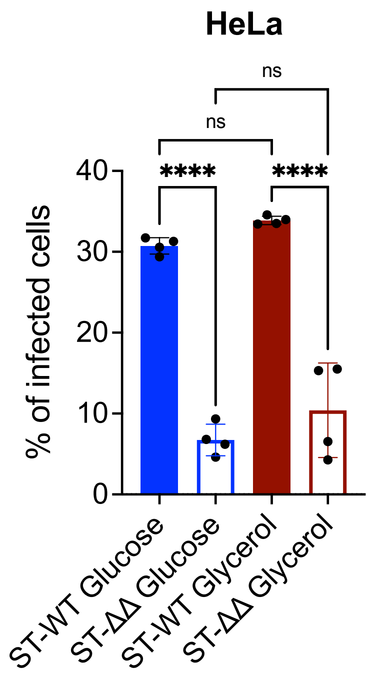

Figure R1. Related to Figure S1G. Percentage of HeLa cells infected by ST-WT or ST-ΔΔ strains when cultured in nutrient-limited medium supplemented with 10 mM glucose or 10 mM glycerol (****=$p<0.0001$, ordinary one-way ANOVA).

3. Care should be taken not to overstate the importance of supplying carbon sources for the replication of *Salmonella*. Although it is true that cells containing replicating *Salmonella* had on average taken up more glucose, it is not a perfect correlation, and it is not true that

"macrophages displaying a higher glucose uptake were those that supported bacterial growth" in line 219.

The Reviewer is right, we have changed the sentence, Line 230: *"Our analyses showed that macrophages supporting bacterial replication of ST-WT and ST-ΔΔ displayed a higher glucose uptake, at times prior to bacterial replication, than non-permissive macrophages, which exhibited lower glucose intake (Figure 4H)"*

4. Given the importance of the live cell imaging analysis in this paper, a more detailed description of the analysis pipeline is necessary in the methods section. Given the amount of noise evident in individual tracks, might it be better to average 2/3 time points from the beginning or end of the track rather than subtracting the first from the last time point to ensure a clear distinction between replicative and non- replicative bacteria.

We thank the Reviewer for this excellent suggestion, which in fact aligns with what our pipeline already does. To determine whether intracellular replication occurred in a given cell, we do not subtract a single first time point from a single last time point. Instead, we average the bacterial area over the first two time points and the last two time points, and compute the difference between these two averages. This approach smooths out the noise from individual measurements and provides a more robust classification of replicative versus non-replicative bacteria, precisely as the Reviewer recommends. This procedure is implemented in our BATLI software, the code for which is publicly available on GitHub (https://github.com/pescoll/BATLI), and was also used in our previous publication where we first described BATLI (Dramé *et al*., Nature Communications, 2025, ref. 25). The implementation can be inspected directly in the BATLI source code (see app.py, line 769 in the code): dA = float(growth[-Np:].mean() - growth[0:Np].mean()), where Np = 2 by default. This computes the difference between the mean bacterial area over the last two time points and the mean over the first two time points, exactly as the reviewer suggests.

We acknowledge that this was insufficiently described in the original Methods section, and we have now expanded the description of the analysis pipeline to make this and other steps explicit. Specifically, the revised Methods section (Line 386 onwards) now includes: *(i)* a detailed description of cell segmentation using Hoechst signal for nuclei and Cell Tracker Blue for cytoplasm boundaries; *(ii)* segmentation of intracellular bacteria using GFP signal; *(iii)* clarification that bacterial replication is assessed by comparing the average bacterial area over the first two time points with the average over the last two time points, with replication defined as an increase exceeding the threshold of 6 µm² for *Salmonella*; *(iv)* a description of single-cell tracking using the overlap-based tracker in Harmony software; and

(v) a reference to the BATLI software for trajectory reconstruction, classification, and backtracking analyses.

We have also changed the legend of Figure 1B, which now reads: *"Replication was considered if the difference between the mean bacterial area over the last two time points and the mean bacterial area over the first two time points exceeded 6 μm^2"*

5. The data of intracellular growth dynamics, e.g. Fig 1D, 2G, 3H, 4C etc are missing stats, which are essential for concluding differences in these datasets. This is also the case for % infected cells with ST in vacuoles/cytoplasm (fig s31 a,b). The authors should take care when describing differences/lack of differences if they are not backed up by the statistics.

We thank the reviewer for this important point. We have now performed comprehensive statistical analyses on all intracellular growth-dynamics panels. To quantify differences in growth trajectories between conditions, we computed the Area Under the Curve (AUC) by trapezoidal integration of the intracellular bacterial area (μm^2) over time for each condition. For panels where per-replicate trajectory data were available (Figures 1D, 2C, and 2G), we performed one-way ANOVA on AUC values across replicates, followed by Tukey HSD post hoc tests for pairwise comparisons. For panels where the source data consist of mean ± SD and N of aggregated single-cell measurements at each timepoint (Figures 3H, 4C, and 4F), we employed a parametric bootstrap approach (B = 10,000 draws, sampling from N(mean, SD) at each timepoint) to generate AUC distributions for each condition, with Bonferroni correction applied to pairwise comparisons. All results are provided in **new Supplementary Table S1**, which contains a summary sheet and individual detail sheets for each panel with per-condition AUC values, global test statistics, and pairwise comparison p-values. The key results are as follows:

<u>Figure 1D</u> (ST-WT in hMDMs across carbon sources): One-way ANOVA on AUC revealed highly significant differences between conditions ($F(3,12) = 44.72$, $p < 0.0001$). Tukey HSD confirmed that glycerol-supplemented macrophages exhibited significantly higher bacterial growth than all other conditions (glycerol vs. glucose $p < 0.0001$; glycerol vs. lactate $p < 0.0001$; glycerol vs. control $p < 0.0001$), while glucose, lactate and control conditions were not significantly different from each other. We have added these statistics to the main text (**line 104**) and figure legend.

<u>Figure 2C</u> (ST-WT growth in hMDMs vs. RAW 264.7): One-way ANOVA on AUC confirmed that ST-WT replicates significantly faster in RAW macrophages than in hMDMs (F(1,6) =

38.51, p = 0.0008). We have added these statistics to the main text (**line 125**) and figure legend.

<u>Figure 2G</u> (ST-WT in RAW macrophages across carbon sources): One-way ANOVA on AUC at the replicate level showed no significant differences between glucose, glycerol, lactate, and control conditions ($F(3,12) = 1.96$, $p = 0.17$). We note that the previous version of the manuscript stated $p < 0.001$ for this comparison, which was based on a single-cell-level analysis with a much larger sample size. We have now revised the text to accurately reflect the replicate-level analysis, which seems more appropriate for this experimental design. Importantly, this non-significant result is consistent with and directly supports our interpretation that ST-WT replication in RAW cells is already near its maximal capacity regardless of the carbon source provided. **Lines 142-145** now read: *"Supplementation with glucose, glycerol or lactate <u>did not significantly alter</u> ST-WT replication rates in permissive RAW cells compared to control conditions (**Figure 2G**, <u>F(3,12) = 1.96, p = 0.17 by one-way ANOVA on AUC, Supplementary Table S1</u>), suggesting that ST-WT replication rate in RAW cells is already near its maximal capacity independently of the supplement used."*

<u>Figure 3H</u> (STvac growth in RAW macrophages across carbon sources): Parametric bootstrap on AUC confirmed significant differences between all three conditions (all pairwise $p < 0.0001$ after Bonferroni correction; glucose AUC = 124.55, glycerol AUC = 107.88, no-C AUC = 83.10). We have added these statistics to the main text (**lines 184-185**) and figure legend.

<u>Figure 4C</u> (ST-WT in hMDMs under 2-DG and glycerol conditions): Bootstrap on AUC confirmed that all four conditions are significantly different from each other (all pairwise $p < 0.0001$ after Bonferroni correction). Notably, we have revised the text at **lines 205-206** to more accurately describe the effects of 2-DG and glycerol supplementation. In particular, we now state that glycerol partially restored ST replication in 2-DG-treated cells, rather than claiming restoration to glucose-fed levels, as the AUC analysis shows that the 2-DG + glycerol condition (AUC = 117.85) remains significantly lower than both glycerol alone (AUC = 178.69) and glucose alone (AUC = 141.00).

<u>Figure 4F</u> (ST-WT in hMDMs across sugars): Bootstrap on AUC revealed a clear hierarchy: glycerol (AUC = 257.73) > galactose (AUC = 225.40) > glucose (AUC = 180.92) > no-C control (AUC = 160.14), with all pairwise comparisons significant ($p < 0.01$ or better after Bonferroni correction, see Supplementary Table S1 for full detail). This ranking reinforces our conclusion that specific carbon sources, rather than glycolytic activity *per se*, drive

intracellular ST replication. We have added these statistics to the main text (**lines 220-222**) and figure legend.

Minor Issues

- Throughout the manuscript the authors refer to infected cells as cells "showing replicative bacteria", more appropriate wording could be containing, harbouring or infected with.

We agree and have changed the wording to "cells harbouring replicative bacteria" throughout the manuscript, including in the legends of Figures 2 and 3, and in lines 19, 569, and 571.

- Could population-level dynamics be provided for data in figure 1H and 2F to compare replicative and non-replicative bacteria (similar to 1D), with appropriate statistics. This could help show a clear difference between these populations and help answer major comment 4.

Please note that Figure 1D excludes non-replicative bacteria and displays only the growth trajectories of replicating bacteria across the different carbon source conditions. The purpose of this plot is to highlight intracellular growth dynamics while minimizing noise from non-replicative bacteria. Figures 1H and 2F serve a different purpose: they display all single-cell trajectories (both replicative and non-replicative) to illustrate the heterogeneity of intracellular bacterial dynamics at the population level. To better distinguish the two subpopulations in these panels, we have now overlaid the median trajectories for the replicating and non-replicating populations as thicker, solid lines on Figures 1B, 1C, 1G, 1H, 2E, and 2F (see also our response to Reviewer #1, point 4). We believe this approach effectively addresses the Reviewer's concern by providing a clear visual comparison of population-level dynamics for each subpopulation without requiring a separate panel.

- Figure S1F requires the nutrient-limited medium control to assess if carbon source supplementation affects *Salmonella* growth.

Figure S1F shows the intracellular growth dynamics (μm^2) of the ST-Δ*prgH*/Δ*ssaV* strain exclusively in those RAW cells that contain replicative bacteria. Under nutrient-limited (no carbon source) conditions, the percentage of cells harbouring replicative ST-Δ*prgH*/Δ*ssaV* bacteria is extremely low (2 ± 1%, as shown in Figure 2I), yielding too few replicative trajectories to compute meaningful growth dynamics. For this reason, a nutrient-limited control curve cannot be included in Figure S1F.

- It is not clear from the figure legend of Fig S1H whether the data is taken from 0 or 20 hpi.

We apologize for the confusion. The data in Figure S1H correspond to the percentage of infected cells at t = 0 hpi (*i.e.*, immediately after infection). We have corrected the figure legend by removing the ambiguous "20 hpi" reference (line 575). The legend now reads: *"Percentage of infected cells at t = 0 hpi, in hMDMs and RAW 264.7 cells, cultured in nutrient-limited medium supplemented with 10 mM glucose and infected with ST-WT or ST-ΔΔ strain"*.

- The representation of data in fig 3B,C,E,F,G,H is very unusual. Why have the authors not used an average and sem rather than showing individual technical repeats?

We chose to display data from individual technical replicates in these panels to transparently illustrate the variability between replicates, which we believe provides a more honest representation of the data than summary statistics alone. Figures 3B, 3C, 3E, 3F, and 3G show the percentage of cells displaying a given phenotype (vacuolar rupture, vacuolar or cytoplasmic localization) over time, measured in four independent replicates from a single experiment representative of three biological replicates. In our experience, showing individual replicate trajectories, rather than a mean ± SEM curve, better conveys the biological variability inherent in these relatively low-frequency events, particularly for the ST-Δ*prgH*/Δ*ssaV* mutant where the percentages are low. However, we acknowledge that this is a choice of data representation, and the underlying data and statistical analyses are provided in the Data Source file and the new **Supplementary Table S1**.

- Can the authors justify how they ensure correct segmentation of bacteria when they are visualising Hoechst, cell tracker and Pvac-tagBFP in the same channel.

This is an important technical point. Hoechst and CellTracker Blue are both excited at 405 nm and detected in the blue channel (435-480 nm emission), but they serve different functions during segmentation: Hoechst provides a strong, concentrated nuclear signal, while CellTracker Blue produces a dimmer, diffuse cytoplasmic signal. These two signals are readily separable by intensity thresholding during image analysis in Harmony software. The nuclei are identified as high-intensity objects and the cytoplasm as the lower-intensity region surrounding them. Pvac-tagBFP, which is expressed by intracellular bacteria within the SCV, also emits in the blue range but is spectrally distinguishable by its slightly red-shifted emission profile and, more importantly, by its spatial localization: Pvac-tagBFP signal is restricted to discrete intracellular spots (bacterial vacuoles) that are morphologically distinct from both nuclear (large, round, high-intensity) and cytoplasmic

(diffuse, low-intensity) signals. Our image analysis pipeline uses a multi-step segmentation approach: 1) nuclei are segmented using the high-intensity Hoechst signal, 2) the cytoplasmic boundary is defined using the lower-intensity CellTracker Blue background, 3) intracellular bacteria are identified as discrete spots based on their size, intensity, and morphological features, using the GFP channel for constitutive bacterial detection and the blue channel for Pvac-tagBFP. Since Pvac-tagBFP is only expressed by vacuolar bacteria and appears as punctate spots with different morphological properties than the diffuse Hoechst/CellTracker signals, this segmentation is robust (see Figure 3D, inset). We have added a clarifying sentence in the Methods section (lines 404-411) describing how the three signals (Hoechst, CellTracker, Pvac-tagBFP) are distinguished using our image analysis pipeline.

- In lines 179-180 the authors conclude that supplementation with glycerol or glucose enhanced cytoplasmic and vacuolar growth but only show data for vacuolar growth.

The reviewer is correct that Figures 3H and 3I show only vacuolar growth data. We have revised lines 179-180, now 183-186, to refer exclusively to vacuolar growth, which is the compartment for which data are presented in these panels. The sentence now reads: *"Supplementation with glycerol or glucose enhanced vacuolar growth of ST-WT compared to control conditions (Figures 3H and 3I, all pairwise comparisons p < 0.0001 by parametric bootstrap on AUC with Bonferroni correction,* ***Supplementary Table S1****), without altering the relative distribution of bacteria between these two compartments (Figure S3A and S3B)."*

- There is no significant difference between control and 2-DG in Fig 4B but this is described as a decrease in lines 198-199. Additionally, glycerol did not restore replication levels to those observed in glucose-fed cells in fig 4C. See major comment 5.

We agree with both observations and have revised the text accordingly. First, for Figure 4B, we no longer describe the difference between glucose and 2-DG conditions as a decrease, since the difference is not statistically significant. Second, regarding Figure 4C, the AUC analysis (**Supplementary Table S1**) confirms that the 2-DG + glycerol condition (AUC = 117.85) remains significantly lower than both glycerol alone (AUC = 178.69) and glucose alone (AUC = 141.00). We have therefore replaced the claim that glycerol "restored" replication to glucose-fed levels with more conservative language. **Lines 202-207** now read: *"At high 2-DG concentration (50 mM), which showed the highest glycolysis inhibition (Figure 4A), the percentage of macrophages with replicative Salmonella was not significantly altered (Figure 4B), although bacterial growth in those cells harbouring*

replicative bacteria was reduced compared to glucose-fed conditions (Figure 4C; p < 0.0001, bootstrap on AUC, Supplementary Table S1). When we provided glycerol to macrophages treated with the high concentration of 2-DG, ST replication was partially restored (Figure 4B and 4C), strongly suggesting that glycolysis is not essential for ST growth in the SCV as long as an alternative carbon source to glucose, such as glycerol, is available."

- Lines 233-235 – this only applies to the T3SS mutant and not the wt *Salmonella*.

We agree. The observation that glucose supplementation showed a gradual, dose-dependent effect on T3SS-independent intracellular replication (Figure S3E and S3F) was specifically measured for the ST-Δ*prgH*/Δ*ssaV* mutant. We have revised lines 245-246 to clarify this: *"Moreover, glucose supplementation showed a gradual effect on T3SS-independent intracellular replication of the* *<u>ST-ΔprgH/ΔssaV mutant</u>* *in RAW macrophages (Figure S3E and S3F), which might have broader implications for reproducibility in infection models."*

- Can the authors explain why they added 2-NBDG and 2-NBF 2 hours after infection. Is it possible that initial infection affected the uptake of sugars?

We added the fluorescent sugar analogs 2-NBDG and 2-NBDF at 2 hours post-infection (hpi), not at the time of infection, for two reasons. First, this timing allowed us to complete the infection protocol (including the 30-minute phagocytosis step, gentamicin washes to eliminate extracellular bacteria, and medium replacement) before introducing the fluorescent probes, ensuring that the probes measured steady-state sugar uptake in stably infected cells rather than transient changes associated with the infection procedure itself. Second, the fluorescent analogs were added at 2 hpi and incubated for 30 minutes, followed by three PBS washes and replacement with fresh medium containing the corresponding unlabelled sugar, meaning that sugar uptake was effectively measured at ~2.5 hpi, early enough to precede the onset of intracellular bacterial replication.

Regarding whether infection itself affects sugar uptake: yes, it does. As shown in Figure 4I, infected macrophages display altered 2-NBDG levels compared to non-infected cells. This is consistent with recent findings showing that glucose uptake in macrophages during *Salmonella* infection is mediated by active upregulation of the glucose transporter GLUT1 (Fang et al, bioRxiv doi: 10.1101/2025.06.10.658782). Importantly, this is precisely what we exploit in our analysis, we correlate the sugar uptake measured at this early timepoint with the subsequent replication outcome of each individual infected cell. Our key finding

(Figure 4H) is that among infected macrophages exposed to identical extracellular glucose concentrations, those that later support bacterial replication are preferentially the ones with higher early glucose uptake, suggesting that it is the metabolic activity of the individual host cell, not merely the extracellular nutrient concentration, that determines intracellular bacterial growth.
