# Supplementary material for: Provision of preferred nutrients to macrophages promotes Salmonella intracellular replication without relying on Type III secretion systems
Source: PLoS Pathog. 2026 Jul 9;22(7):e1014348. doi: 10.1371/journal.ppat.1014348 (PMC13349091; doi:10.1371/journal.ppat.1014348)
Supplement: Attachment — Submitted filename: point-by-point-answers_auresp_3.pdf [file ppat.1014348.s009.pdf]

POINT-BY-POINT ANSWER TO EDITOR

Editor

The data you provide for reviewer's only (Figure R1) is actually important for all readers to see, as based on 30 years of Salmonella research, a T3SS1 mutant should have a drastic defect in invasion of HeLa cells. At the population level (gentamicin assay) there is a 2-3 log fold reduction in invasion for a T3SS mutant in HeLa cells. Measuring % of infected cells is misleading because it suggests there is only a 6-fold reduction in bacterial invasion (30% infected cells down to 5%) but I suspect there is a vast difference in the number of bacteria that have been internalized for WT and the double mutant. There has to be a difference in the number of internalized bacteria in the absence of a functional PrgH in your infection conditions. If not, you should independently confirm that your prgH mutant is T3SS1 defective. Please include data (as Supplemental) fully characterizing the double mutant for invasion of HeLa cells (WT versus prgHssaV mutant) - this would include gentamicin protection assay, current provided data (% infected cells) and also the mean number of bacteria in each infected cell. This is very easy data to generate and you may already have it on-hand. As your findings are challenging dogma, I believe providing this full characterization is essential to assuage any concerns that readers may have about the validity of this bacterial mutant.

We thank the Editor for this constructive comment. We fully agree that, given that our findings challenge a long-standing paradigm, a complete characterization of the invasion phenotype of the Δ*prgH*/Δ*ssaV* double mutant in HeLa cells is essential for readers to be confident in the validity of the strain. We have now performed the requested experiments and added them to the manuscript. We have generated a **new supplementary Figure S2** that fully characterizes the invasion of HeLa cells by ST-WT versus the ST-Δ*prgH*/Δ*ssaV* double mutant, using the three complementary readouts requested by the Editor:

(A) the percentage of infected HeLa cells (automated confocal microscopy); (B) the total number of intracellular bacteria per well (microscopy); (C) the percentage of invasion measured by gentamicin protection assay (intracellular CFU at t0 / inoculum); and (D) the total number of intracellular bacteria per well measured by gentamicin protection assay (CFU at t0 per well).

As the Editor correctly anticipated, the gentamicin protection assay reveals a drastic invasion defect of the double mutant. At the population level (**S2C** and **S2D**), invasion of HeLa cells by the ST-Δ*prgH*/Δ*ssaV* mutant is reduced by approximately three orders of magnitude relative to ST-WT (~6.5% invasion and ~5.6 × 10^6 intracellular CFU per well for

ST-WT versus ~0.009% invasion and ~8 × 10^3 CFU per well for the double mutant in glucose), entirely consistent with the expected behavior of a T3SS1-deficient strain that cannot efficiently invade non-phagocytic epithelial cells. This independently confirms that our ST-Δ*prgH*/Δ*ssaV* mutant is genuinely T3SS1-defective.

We also agree with the Editor's important observation that the percentage of infected cells (**S2A**) substantially underestimates this defect. As the Editor suspected, the apparent ~6-fold reduction in the percentage of infected cells (from 30.7% to 6.7% in glucose, **S2A**) does not reflect the true difference in the number of internalized bacteria. The microscopy-based total bacterial burden per well (**S2B**) shows a markedly larger reduction, and the gentamicin protection assay (**S2C and D**) shows the full ~3-log difference in internalized bacteria.

To accompany these data, we have added a call to the new figure in the Results (**lines 156-157**), which reads: *"The drastic invasion defect of the T3SS-deficient double mutant in HeLa cells was confirmed by gentamicin protection assay (**Figure S2**)".*

We also added a dedicated Methods subsection, "Gentamicin protection assay for invasion of HeLa cells" (**lines 428-439**).

We are confident that this full characterization addresses the Editor's concern and removes any ambiguity about the identity and T3SS1-deficiency of the double mutant.
