# Supplementary material for: Provision of preferred nutrients to macrophages promotes Salmonella intracellular replication without relying on Type III secretion systems
Source: PLoS Pathog. 2026 Jul 9;22(7):e1014348. doi: 10.1371/journal.ppat.1014348 (PMC13349091; doi:10.1371/journal.ppat.1014348)
Supplement: Peer review history

# Author comment: Author response to Decision Letter 0

**DOI:** 10.1371/journal.ppat.1014348.r001

20 Oct 2025

Attachment Submitted filename: Revision-plan-RC-2025-03098.pdf

# Editor report: Decision Letter 0

**DOI:** 10.1371/journal.ppat.1014348.r002

**Authors:** Sophie Helaine, Eva Heinz

27 Oct 2025

-->PPATHOGENS-D-25-02595

Provision of Preferred Nutrients to Macrophages Enables Salmonella to Replicate Intracellularly Without Relying on Type III Secretion Systems

PLOS Pathogens

Dear Dr. Escoll,

Thank you for submitting your manuscript to PLOS Pathogens. After careful consideration, we feel that it has merit but does not fully meet PLOS Pathogens's publication criteria as it currently stands. Therefore, we invite you to submit a revised version of the manuscript that addresses the points raised during the review process.

Please submit your revised manuscript within 60 days Dec 26 2025 11:59PM. If you will need more time than this to complete your revisions, please reply to this message or contact the journal office at plospathogens@plos.org. When you're ready to submit your revision, log on to https://www.editorialmanager.com/ppathogens/ and select the 'Submissions Needing Revision' folder to locate your manuscript file.

Please include the following items when submitting your revised manuscript:

* A rebuttal letter that responds to each point raised by the editor and reviewer(s). You should upload this letter as a separate file labeled 'Response to Reviewers'. This file does not need to include responses to any formatting updates and technical items listed in the 'Journal Requirements' section below.

* A marked-up copy of your manuscript that highlights changes made to the original version. You should upload this as a separate file labeled 'Revised Manuscript with Track Changes'.

* An unmarked version of your revised paper without tracked changes. You should upload this as a separate file labeled 'Manuscript'.

If you would like to make changes to your financial disclosure, competing interests statement, or data availability statement, please make these updates within the submission form at the time of resubmission. Guidelines for resubmitting your figure files are available below the reviewer comments at the end of this letter.

We look forward to receiving your revised manuscript.

Kind regards,

Eva Heinz

Section Editor

PLOS Pathogens

Sumita Bhaduri-McIntosh

Editor-in-Chief

PLOS Pathogens

orcid.org/0000-0003-2946-9497

Michael Malim

Editor-in-Chief

PLOS Pathogens

orcid.org/0000-0002-7699-2064

**Additional Editor Comments :**

We have carefully read the reviews from Review Commons and would like to invite you to prepare a revised version of the manuscript accordingly. Just so you know, the revised manuscript will have to be sent back, ideally, to the same two reviewers and a new one for assessment.

**Journal Requirements:**

1) We ask that a manuscript source file is provided at Revision. Please upload your manuscript file as a .doc, .docx, .rtf or .tex. If you are providing a .tex file, please upload it under the item type u2018LaTeX Source Fileu2019 and leave your .pdf version as the item type u2018Manuscriptu2019.

2) Please provide an Author Summary. This should appear in your manuscript between the Abstract (if applicable) and the Introduction, and should be 150-200 words long. The aim should be to make your findings accessible to a wide audience that includes both scientists and non-scientists. Sample summaries can be found on our website under Submission Guidelines:

https://journals.plos.org/plospathogens/s/submission-guidelines#loc-parts-of-a-submission

3) Your manuscript is missing the following section heading: Abstract.  Please ensure all required sections are present and in the correct order. Make sure section heading levels are clearly indicated in the manuscript text, and limit sub-sections to 3 heading levels. An outline of the required sections can be consulted in our submission guidelines here:

https://journals.plos.org/plospathogens/s/submission-guidelines#loc-parts-of-a-submission

4) Thank you for including an Ethics Statement for your study. Please include:

i) The full name(s) of the Institutional Review Board(s) or Ethics Committee(s).

5) Please upload all main figures as separate Figure files in .tif or .eps format. For more information about how to convert and format your figure files please see our guidelines:

https://journals.plos.org/plospathogens/s/figures

6) We notice that your supplementary Figures are included in the manuscript file. Please remove them and upload them with the file type 'Supporting Information'. Please ensure that each Supporting Information file has a legend listed in the manuscript after the references list.

7) We note that your Data Availability Statement is currently as follows: "All relevant data are within the manuscript and its Supporting Information files". Please confirm at this time whether or not your submission contains all raw data required to replicate the results of your study. Authors must share the “minimal data set” for their submission. PLOS defines the minimal data set to consist of the data required to replicate all study findings reported in the article, as well as related metadata and methods (https://journals.plos.org/plosone/s/data-availability#loc-minimal-data-set-definition).

For example, authors should submit the following data:

1) The values behind the means, standard deviations and other measures reported;

2) The values used to build graphs;

3) The points extracted from images for analysis..

Authors do not need to submit their entire data set if only a portion of the data was used in the reported study.

If your submission does not contain these data, please either upload them as Supporting Information files or deposit them to a stable, public repository and provide us with the relevant URLs, DOIs, or accession numbers. For a list of recommended repositories, please see https://journals.plos.org/plosone/s/recommended-repositories.

If there are ethical or legal restrictions on sharing a de-identified data set, please explain them in detail (e.g., data contain potentially sensitive information, data are owned by a third-party organization, etc.) and who has imposed them (e.g., an ethics committee). Please also provide contact information for a data access committee, ethics committee, or other institutional body to which data requests may be sent. If data are owned by a third party, please indicate how others may request data access.

8) Please amend your detailed Financial Disclosure statement. This is published with the article. It must therefore be completed in full sentences and contain the exact wording you wish to be published.

1) State what role the funders took in the study. If the funders had no role in your study, please state: "The funders had no role in study design, data collection and analysis, decision to publish, or preparation of the manuscript.".

Note: If the reviewer comments include a recommendation to cite specific previously published works, please review and evaluate these publications to determine whether they are relevant and should be cited. There is no requirement to cite these works unless the editor has indicated otherwise.

**Figure resubmission:**

-->While revising your submission, we strongly recommend that you use PLOS’s NAAS tool (https://ngplosjournals.pagemajik.ai/artanalysis) to test your figure files. NAAS can convert your figure files to the TIFF file type and meet basic requirements (such as print size, resolution), or provide you with a report on issues that do not meet our requirements and that NAAS cannot fix.

After uploading your figures to PLOS’s NAAS tool - https://ngplosjournals.pagemajik.ai/artanalysis, NAAS will process the files provided and display the results in the "Uploaded Files" section of the page as the processing is complete. If the uploaded figures meet our requirements (or NAAS is able to fix the files to meet our requirements), the figure will be marked as "fixed" above. If NAAS is unable to fix the files, a red "failed" label will appear above. When NAAS has confirmed that the figure files meet our requirements, please download the file via the download option, and include these NAAS processed figure files when submitting your revised manuscript.

**Reproducibility:**

To enhance the reproducibility of your results, we recommend that authors of applicable studies deposit laboratory protocols in protocols.io, where a protocol can be assigned its own identifier (DOI) such that it can be cited independently in the future. Additionally, PLOS ONE offers an option to publish peer-reviewed clinical study protocols. Read more information on sharing protocols at https://plos.org/protocols?utm_medium=editorial-email&utm_source=authorletters&utm_campaign=protocols

# Author comment: Author response to Decision Letter 1

**DOI:** 10.1371/journal.ppat.1014348.r003

28 Jan 2026

Attachment Submitted filename: point-by-point-answers.pdf

# Peer review: Decision Letter 1

**DOI:** 10.1371/journal.ppat.1014348.r004

**Authors:** Sophie Helaine, Eva Heinz, Sophie Helaine, Eva Heinz

16 Mar 2026

PPATHOGENS-D-25-02595R1

Provision of Preferred Nutrients to Macrophages Enables Salmonella to Replicate Intracellularly Without Relying on Type III Secretion Systems

PLOS Pathogens

Dear Dr. Escoll,

Thank you for submitting your manuscript to PLOS Pathogens. Two reviewers have assessed the manuscript, including the previous reviews and answers provided by Review Commons. Reviewers find the work valuable, and we are interested in publishing the work. However, it requires revisions. Reviewers are both adamant that rewording is required and the conclusions need to match the results more closely, including the title. In addition, both reviewers flag infections of HeLa cells with a double spi1 spi2 mutant as problematic. We would like you to address the comments in full, mostly through text edits, statistical analyses, and edits to the representation of the data, rather than through additional experimental work.

Please submit your revised manuscript by May 15 2026 11:59PM. If you will need more time than this to complete your revisions, please reply to this message or contact the journal office at plospathogens@plos.org. When you're ready to submit your revision, log on to https://www.editorialmanager.com/ppathogens/ and select the 'Submissions Needing Revision' folder to locate your manuscript file.

Please include the following items when submitting your revised manuscript:

* A letter that responds to each point raised by the editor and reviewer(s). You should upload this letter as a separate file labeled 'Response to Reviewers'. This file does not need to include responses to any formatting updates and technical items listed in the 'Journal Requirements' section below.

* A marked-up copy of your manuscript that highlights changes made to the original version. You should upload this as a separate file labeled 'Revised Manuscript with Track Changes'.

* An unmarked version of your revised paper without tracked changes. You should upload this as a separate file labeled 'Manuscript'.

If you would like to make changes to your financial disclosure, competing interests statement, or data availability statement, please make these updates within the submission form at the time of resubmission. Guidelines for resubmitting your figure files are available below the reviewer comments at the end of this letter.

We look forward to receiving your revised manuscript.

Kind regards,

Sophie Helaine

Academic Editor

PLOS Pathogens

Eva Heinz

Section Editor

PLOS Pathogens

Sumita Bhaduri-McIntosh

Editor-in-Chief

PLOS Pathogens

orcid.org/0000-0003-2946-9497

Michael Malim

Editor-in-Chief

PLOS Pathogens

orcid.org/0000-0002-7699-2064

**Journal Requirements:**

If the reviewer comments include a recommendation to cite specific previously published works, please review and evaluate these publications to determine whether they are relevant and should be cited. There is no requirement to cite these works unless the editor has indicated otherwise.

1) Please amend your detailed Financial Disclosure statement. This is published with the article. It must therefore be completed in full sentences and contain the exact wording you wish to be published.

1) State the initials, alongside each funding source, of each author to receive each grant. For example: "This work was supported by the National Institutes of Health (####### to AM; ###### to CJ) and the National Science Foundation (###### to AM)."

2) State what role the funders took in the study. If the funders had no role in your study, please state: "The funders had no role in study design, data collection and analysis, decision to publish, or preparation of the manuscript."

3) If any authors received a salary from any of your funders, please state which authors and which funders..

If you did not receive any funding for this study, please simply state: u201cThe authors received no specific funding for this work.u201d

**Reviewers' Comments:**

Reviewer's Responses to Questions

**Part I - Summary**

Please use this section to discuss strengths/weaknesses of study, novelty/significance, general execution and scholarship.

Reviewer #1: In this study, the authors explore how variability in host cell metabolism shapes the intracellular growth of Salmonella enterica serovar Typhimurium. Using live-cell imaging in infected human primary macrophages, they show that bacterial replication is unevenly distributed, occurring only in a subset of infected cells. They find that adding particular carbon sources—known to be utilized by Salmonella during infection—enhances bacterial proliferation and increases the fraction of macrophages that permit intracellular replication. Notably, this stimulatory effect is also observed in a ΔprgH/ΔssaV double mutant lacking functional Type III Secretion Systems (T3SS). The authors propose that the boost in replication is not primarily driven by host glycolysis itself, but instead depends on the host cell’s capacity to take up nutrients. Overall, the data suggest that under nutrient-rich conditions, intracellular Salmonella can capitalize on host metabolic resources to replicate, even in the absence of its classical virulence-associated secretion systems.

Reviewer #2: During infection Salmonella resides within a host membrane-bound compartment called the Salmonella-containing vacuole (SCV). The SCV is a restrictive environment for Salmonella growth. One of the roles of the SPI-2 T3SS is to modify the SCV to enable intracellular replication. Here the authors show that intracellular replication is enhanced by the availability of carbon sources in the host cell media, and this is dependent on the ability of Salmonella to metabolise these carbon sources. Really interestingly, they show that providing host cells with supplementary carbon sources can even support the growth of T3SS mutants in a subset of cells. However, the authors need to take care (i) not to overstate these findings and (ii) to only make conclusions that are backed up by statistically significant data.

**********

**Part II – Major Issues: Key Experiments Required for Acceptance**

Please use this section to detail the key new experiments or modifications of existing experiments that should be absolutely required to validate study conclusions.

Generally, there should be no more than 3 such required experiments or major modifications for a "Major Revision" recommendation. If more than 3 experiments are necessary to validate the study conclusions, then you are encouraged to recommend "Reject".

Reviewer #1: 1. This reviewer finds the title highly misleading, in the sense it implies the absence of T3SSs can be fully compensated by the right nutrient supplementation. The authors demonstrate only partial restoration of intracellular replication with nutrient supplementation, and only a fraction of that compared to T3SS-expressing STm. Unless the authors can demonstrate complete replication competence comparable to WT STm with nutrient supplementation, the authors should tone down the phrasing of the title, as was acknowledged and done by the authors based on the former reviewers’ requests. To argue that the T3SS is indispensable for intracellular STm growth is not supported by the evidence presented in the manuscript.

2. Authors should exercise more caution in their interpretation and phrasing. For example, this reviewer strongly recommends to rephrase wording in the abstract and throughout, such as L29: ‘When equivalent metabolic conditions are achieved by other means, dependence on T3SSs becomes dispensable’ to ‘dependence on T3SSs for intracellular replication can be partially compensated in vitro’. STm T3SS mutants (SPI-1 and/or SPI-2) are highly attenuated for virulence in in vitro and in animal models. This reviewer remains sceptical as to whether any amount of nutrient supplementation in any in vitro or in vivo model (while the latter are admittedly are challenging experiments), is sufficient to fully compensate for the absence of one or both SPI-1/2 T3SSs. I therefore encourage the authors to exercise more caution in suggesting that the T3SS is dispensable, as long as the right nutrients are provided. At best what the authors show is that it partially restores intracellular replication of a STm-T3SS-deficient mutant in vitro. There is no evidence yet to demonstrate this effect is meaningful for virulence/pathogenesis in an infection scenario.

3. Figure S1G seems to be missing a control, only glycerol and glucose conditions are shown. Also, the experiment in HeLa cells should not be performed with a double mutant, rather a SPI-2 mutant only, as a SPI-1 deficient STm will not allow invasion into HeLa cells at all, an thus have no infected cells to measure. Thus, such an experiment should be performed with a single SPI-2 mutant.

Reviewer #2: 1. Effectors of the SPI-2 T3SS have multiple roles during systemic infection, including disrupting the host immune response. This should be made clear. Carbon source supplementation does not restore intracellular replication of T3SS mutants to the level of wt Salmonella. Therefore, suggestions that nutrient can “override the requirement of the T3SS”, or that the “primary purposes of T3SS effector proteins is to overcome host-imposed nutrient restriction within macrophage vacuoles” should be toned down, including in lines 41, 264, 247, line 288 and line 306.

a. It would be interesting to see if the replication defect of the SseFG deletion can be rescued by nutrient availability.

2. The authors stress this as a macrophage-specific response. In response to previous reviewer #1 they have checked the phenotype in HeLa cells. Although this data in Fig S1G shows no difference between cells supplemented with glycerol or glucose, it doesn’t include the control grown in nutrient limited media. Data from HeLa cells on the intracellular growth dynamics of replicative bacteria is also absent. It is therefore impossible to judge whether supplementation of HeLa cells with carbon sources affects Salmonella replication. Without this data, the authors should remove arguments that this is a macrophage specific effect. Additionally, Salmonella relies on the SPI-1 T3SS for infection of epithelial cell lines. It is therefore likely that the double T3SS mutant has very low infection rates in HeLa cells. It may therefore be better to use ∆ssaV mutants for these experiments.

3. Care should be taken not to overstate the importance of supplying carbon sources for the replication of Salmonella. Although it is true that cells containing replicating Salmonella had on average taken up more glucose, it is not a perfect correlation and it is not true that “macrophages displaying a higher glucose uptake were those that supported bacterial growth” in line 219.

4. Given the importance of the live cell imaging analysis in this paper, a more detailed description of the analysis pipeline is necessary in the methods section. Given the amount of noise evident in individual tracks, might it be better to average 2/3 time points from the beginning or end of the track rather than subtracting the first from the last time point to ensure a clear distinction between replicative and non-replicative bacteria.

5. The data of intracellular growth dynamics, eg Fig 1D, 2G, 3H, 4C etc are missing stats, which are essential for concluding differences in these datasets. This is also the case for % infected cells with ST in vacuoles/cytoplasm (fig s31 a,b). The authors should take care when describing differences/lack of differences if they are not backed up by the statistics.

**********

**Part III – Minor Issues: Editorial and Data Presentation Modifications**

Please use this section for editorial suggestions as well as relatively minor modifications of existing data that would enhance clarity.

Reviewer #1: 4. Figure 1. B, C, G ,H. The number of infected cells analysed in B, C, G and H need to be similar. Otherwise the traces become misleading. Currently C shows 187 single cell trajectories, whereas H, stands at 332. As these two conditions should be compared directly, it is important to have similar cell numbers. The authors could perform random sampling for each of the groups with a unified total number of cells per group. This will enable fairer comparison between the four groups and indication of the effect size the authors are observe compared to WT conditions, and double mutant +/- glycerol. The authors should also overlay a smoothed average for each ‘replicating(red)’ and ‘non-replicating(blue)’ population to better illustrate the population averages of these two subpopulations of infected cells over time.

5. Figure 1. F. The Y-axis in Figure 1F should be set to 100%, as it is on 1E. This is important for readers to gauge the size of the effects observed relative to WT conditions.

6. Figure S3F x- axis should be made to 100% for a fairer comparison to Figure S2E

7. The authors claim it is the metabolic niche of the host that dictates T3SS-independent intracellular proliferation. However, as the nutrient supplementations being tested are added exogenously in tissue culture media, the effect of the carbon source is via supplemented into the media, and since macrophages are constantly sampling and engulfing nutrients, the hosts’ role in this process is probably more passive than what is suggested by the authors. Where the host is probably doing nothing more than passing the nutrients to STm via active and continuous engulfment and internalisation of extracellular media components, rather than the nutrients STm is exposed to in the SCV being the result of any direct and active shaping/modulation by the host per se. This is an important distinction, as it relates to the question as to whether the effect observed by the authors is a phenomenon that is only observable in in tissue culture experiments. An observation that this phenomenon is also observed in non-phagocytic cells (e.g. HeLa) would argue in favor of the authors suggestion that the host plays a more active role, whereas if this phenomenon is only observed in phagocytic cells it could be a more passive effect of STm and media components being brought into close proximity within the endosomal compartment of infected cells. This relates to experiments suggested by former previous reviewer 1 and the suggested experiment above with the single SPI-2 mutant (minor point 5).

Reviewer #2: - Throughout the manuscript the authors refer to infected cells as cells “showing replicative bacteria”, more appropriate wording could be containing, harbouring or infected with.

- Could population-level dynamics be provided for data in figure 1H and 2F to compare replicative and non-replicative bacteria (similar to 1D), with appropriate statistics. This could help show a clear difference between these populations and help answer major comment 4.

- Figure S1F requires the nutrient-limited medium control to assess if carbon source supplementation affects Salmonella growth.

- It is not clear from the figure legend of Fig S1H whether the data is taken from 0 or 20 hpi.

- The representation of data in fig 3B,C,E,F,G,H is very unusual. Why have the authors not used an average and sem rather than showing individual technical repeats?

- Can the authors justify how they ensure correct segmentation of bacteria when they are visualising Hoechst, cell tracker and Pvac-tagBFP in the same channel.

- In lines 179-180 the authors conclude that supplementation with glycerol or glucose enhanced cytoplasmic and vacuolar growth but only show data for vacuolar growth.

- There is no significant difference between control and 2-DG in Fig 4B but this is described as a decrease in lines 198-199. Additionally, glycerol did not restore replication levels to those observed in glucose-fed cells in fig 4C. See major comment 5.

- Lines 233-235 – this only applies to the T3SS mutant and not the wt Salmonella.

- Can the authors explain why they added 2-NBDG and 2-NBF 2 hours after infection. Is it possible that initial infection affected the uptake of sugars?

**********

PLOS authors have the option to publish the peer review history of their article (what does this mean?). If published, this will include your full peer review and any attached files.

If you choose “no”, your identity will remain anonymous but your review may still be made public.

**Do you want your identity to be public for this peer review?**  For information about this choice, including consent withdrawal, please see our Privacy Policy.

Reviewer #1: No

Reviewer #2: No

**Figure resubmission:**

While revising your submission, we strongly recommend that you use PLOS’s NAAS tool (https://ngplosjournals.pagemajik.ai/artanalysis) to test your figure files. NAAS can convert your figure files to the TIFF file type and meet basic requirements (such as print size, resolution), or provide you with a report on issues that do not meet our requirements and that NAAS cannot fix.

After uploading your figures to PLOS’s NAAS tool - https://ngplosjournals.pagemajik.ai/artanalysis, NAAS will process the files provided and display the results in the "Uploaded Files" section of the page as the processing is complete. If the uploaded figures meet our requirements (or NAAS is able to fix the files to meet our requirements), the figure will be marked as "fixed" above. If NAAS is unable to fix the files, a red "failed" label will appear above. When NAAS has confirmed that the figure files meet our requirements, please download the file via the download option, and include these NAAS processed figure files when submitting your revised manuscript.

**Reproducibility:**

To enhance the reproducibility of your results, we recommend that authors of applicable studies deposit laboratory protocols in protocols.io, where a protocol can be assigned its own identifier (DOI) such that it can be cited independently in the future. Additionally, PLOS ONE offers an option to publish peer-reviewed clinical study protocols. Read more information on sharing protocols at https://plos.org/protocols?utm_medium=editorial-email&utm_source=authorletters&utm_campaign=protocols

# Author comment: Author response to Decision Letter 2

**DOI:** 10.1371/journal.ppat.1014348.r005

23 Apr 2026

Attachment Submitted filename: point-by-point-answers_auresp_2.pdf

# Editor report: Decision Letter 2

**DOI:** 10.1371/journal.ppat.1014348.r006

**Authors:** Sophie Helaine, Eva Heinz, Sophie Helaine, Eva Heinz, Leigh Knodler, Eva Heinz

10 May 2026

PPATHOGENS-D-25-02595R2

Provision of Preferred Nutrients to Macrophages Promotes Salmonella Intracellular Replication Without Relying on Type III Secretion Systems

PLOS Pathogens

Dear Dr. Escoll,

Thank you for submitting your manuscript to PLOS Pathogens. After careful consideration, we feel that it has merit but does not fully meet PLOS Pathogens's publication criteria as it currently stands. Therefore, we invite you to submit a revised version of the manuscript that addresses the points raised during the review process.

Please submit your revised manuscript by Jul 09 2026 11:59PM. If you will need more time than this to complete your revisions, please reply to this message or contact the journal office at plospathogens@plos.org. When you're ready to submit your revision, log on to https://www.editorialmanager.com/ppathogens/ and select the 'Submissions Needing Revision' folder to locate your manuscript file.

Please include the following items when submitting your revised manuscript:

* A letter that responds to each point raised by the editor and reviewer(s). You should upload this letter as a separate file labeled 'Response to Reviewers'. This file does not need to include responses to any formatting updates and technical items listed in the 'Journal Requirements' section below.

* A marked-up copy of your manuscript that highlights changes made to the original version. You should upload this as a separate file labeled 'Revised Manuscript with Track Changes'.

* An unmarked version of your revised paper without tracked changes. You should upload this as a separate file labeled 'Manuscript'.

If you would like to make changes to your financial disclosure, competing interests statement, or data availability statement, please make these updates within the submission form at the time of resubmission. Guidelines for resubmitting your figure files are available below the reviewer comments at the end of this letter.

We look forward to receiving your revised manuscript.

Kind regards,

Leigh Knodler

Academic Editor

PLOS Pathogens

Eva Heinz

Section Editor

PLOS Pathogens

Sumita Bhaduri-McIntosh

Editor-in-Chief

PLOS Pathogens

orcid.org/0000-0003-2946-9497

Michael Malim

Editor-in-Chief

PLOS Pathogens

orcid.org/0000-0002-7699-2064

**Editor Comments:**

Dear Pedro,

Thank you for submitting a revised version of your manuscript.

The data you provide for reviewer's only (Figure R1) is actually important for all readers to see, as based on 30 years of Salmonella research, a T3SS1 mutant should have a drastic defect in invasion of HeLa cells. At the population level (gentamicin assay) there is a 2-3 log fold reduction in invasion for a T3SS mutant in HeLa cells. Measuring % of infected cells is misleading because it suggests there is only a 6-fold reduction in bacterial invasion (30% infected cells down to 5%) but I suspect there is a vast difference in the number of bacteria that have been internalized for WT and the double mutant. There has to be a difference in the number of internalized bacteria in the absence of a functional PrgH in your infection conditions. If not, you should independently confirm that your prgH mutant is T3SS1 defective. Please include data (as Supplemental) fully characterizing the double mutant for invasion of HeLa cells (WT versus prgHssaV mutant) - this would include gentamicin protection assay, current provided data (% infected cells) and also the mean number of bacteria in each infected cell. This is very easy data to generate and you may already have it on-hand. As your findings are challenging dogma, I believe providing this full characterization is essential to assuage any concerns that readers may have about the validity of this bacterial mutant.

Best wishes, Leigh

**Figure resubmission:**

-->While revising your submission, we strongly recommend that you use PLOS’s NAAS tool (https://ngplosjournals.pagemajik.ai/artanalysis) to test your figure files. NAAS can convert your figure files to the TIFF file type and meet basic requirements (such as print size, resolution), or provide you with a report on issues that do not meet our requirements and that NAAS cannot fix.

After uploading your figures to PLOS’s NAAS tool - https://ngplosjournals.pagemajik.ai/artanalysis, NAAS will process the files provided and display the results in the "Uploaded Files" section of the page as the processing is complete. If the uploaded figures meet our requirements (or NAAS is able to fix the files to meet our requirements), the figure will be marked as "fixed" above. If NAAS is unable to fix the files, a red "failed" label will appear above. When NAAS has confirmed that the figure files meet our requirements, please download the file via the download option, and include these NAAS processed figure files when submitting your revised manuscript.

**Reproducibility:**

To enhance the reproducibility of your results, we recommend that authors of applicable studies deposit laboratory protocols in protocols.io, where a protocol can be assigned its own identifier (DOI) such that it can be cited independently in the future. Additionally, PLOS ONE offers an option to publish peer-reviewed clinical study protocols. Read more information on sharing protocols at https://plos.org/protocols?utm_medium=editorial-email&utm_source=authorletters&utm_campaign=protocols

# Author comment: Author response to Decision Letter 3

**DOI:** 10.1371/journal.ppat.1014348.r007

30 May 2026

Attachment Submitted filename: point-by-point-answers_auresp_3.pdf

# Editor report: Decision Letter 3

**DOI:** 10.1371/journal.ppat.1014348.r008

**Authors:** Sophie Helaine, Eva Heinz, Sophie Helaine, Eva Heinz, Leigh Knodler, Eva Heinz, Sophie Helaine, Eva Heinz

3 Jun 2026

Dear Dr Escoll,

We are pleased to inform you that your manuscript 'Provision of Preferred Nutrients to Macrophages Promotes Salmonella Intracellular Replication Without Relying on Type III Secretion Systems' has been provisionally accepted for publication in PLOS Pathogens.

Before your manuscript can be formally accepted you will need to complete some formatting changes, which you will receive in a follow up email. A member of our team will be in touch with a set of requests.

Please note that your manuscript will not be scheduled for publication until you have made the required changes, so a swift response is appreciated.

IMPORTANT: The editorial review process is now complete. PLOS will only permit corrections to spelling, formatting or significant scientific errors from this point onwards. Requests for major changes, or any which affect the scientific understanding of your work, will cause delays to the publication date of your manuscript.

Should you, your institution's press office or the journal office choose to press release your paper, you will automatically be opted out of early publication. We ask that you notify us now if you or your institution is planning to press release the article. All press must be co-ordinated with PLOS.

Thank you again for supporting Open Access publishing; we are looking forward to publishing your work in PLOS Pathogens.

Best regards,

Sophie Helaine

Academic Editor

PLOS Pathogens

Eva Heinz

Section Editor

PLOS Pathogens

Sumita Bhaduri-McIntosh

Editor-in-Chief

PLOS Pathogens

orcid.org/0000-0003-2946-9497

Michael Malim

Editor-in-Chief

PLOS Pathogens

orcid.org/0000-0002-7699-2064

***********************************************************

Thank you for your extensive efforts in revising this manuscript. We are very pleased to accept it.

Reviewer Comments (if any, and for reference):

# Editor report: Acceptance letter

**DOI:** 10.1371/journal.ppat.1014348.r009

**Authors:** Sophie Helaine, Eva Heinz, Sophie Helaine, Eva Heinz, Leigh Knodler, Eva Heinz, Sophie Helaine, Eva Heinz

Dear Dr Escoll,

We are delighted to inform you that your manuscript, "Provision of Preferred Nutrients to Macrophages Promotes Salmonella Intracellular Replication Without Relying on Type III Secretion Systems," has been formally accepted for publication in PLOS Pathogens.

We have now passed your article onto the PLOS Production Department who will complete the rest of the pre-publication process. All authors will receive a confirmation email upon publication.

The corresponding author will soon be receiving a typeset proof for review, to ensure errors have not been introduced during production. Please review the PDF proof of your manuscript carefully, as this is the last chance to correct any scientific or type-setting errors. Please note that major changes, or those which affect the scientific understanding of the work, will likely cause delays to the publication date of your manuscript. Note: Proofs for Front Matter articles (Pearls, Reviews, Opinions, etc...) are generated on a different schedule and may not be made available as quickly.

Soon after your final files are uploaded, the early version of your manuscript, if you opted to have an early version of your article, will be published online. The date of the early version will be your article's publication date. The final article will be published to the same URL, and all versions of the paper will be accessible to readers.

For Research Articles, you will receive an invoice from PLOS for your publication fee after your manuscript has reached the completed accept phase. If you receive an email requesting payment before acceptance or for any other service, this may be a phishing scheme. Learn how to identify phishing emails and protect your accounts at https://explore.plos.org/phishing.

Thank you again for supporting open-access publishing; we are looking forward to publishing your work in PLOS Pathogens.

Best regards,

Sumita Bhaduri-McIntosh

Editor-in-Chief

PLOS Pathogens

orcid.org/0000-0003-2946-9497

Michael Malim

Editor-in-Chief

PLOS Pathogens

orcid.org/0000-0002-7699-2064
